# Recommended Implementation of Quantitative Susceptibility Mapping for Clinical Research in The Brain: A Consensus of the ISMRM Electro-Magnetic Tissue Properties Study Group

**Published:** 2023-07-05

**Authors:** QSM Consensus Organization Committee, Berkin Bilgic, Mauro Costagli, Kwok-Shing Chan, Jeff Duyn, Christian Langkammer, Jongho Lee, Xu Li, Chunlei Liu, José P. Marques, Carlos Milovic, Simon Robinson, Ferdinand Schweser, Karin Shmueli, Pascal Spincemaille, Sina Straub, Peter van Zijl, Yi Wang, ISMRM Electro-Magnetic Tissue Properties Study Group

**Affiliations:** 1 – Martinos Center for Biomedical Imaging, Massachusetts General Hospital and Harvard Medical School, Charlestown, MA, United States; 2 – Department of Neuroscience, Rehabilitation, Ophthalmology, Genetics, Maternal and Child Sciences (DINOGMI), University of Genoa, Genoa, Italy; 3 – Laboratory of Medical Physics and Magnetic Resonance, IRCCS Stella Maris, Pisa, Italy; 4 – Donders Institute for Brain, Cognition and Behaviour, Radboud University, Nijmegen, the Netherlands; 5 – Advanced MRI Section, NINDS, National Institutes of Health, Bethesda, MD, United States; 6 – Department of Neurology, Medical University of Graz, Graz, Austria; 7 – Department of Electrical and Computer Engineering, Seoul National University, Seoul, Republic of Korea; 8 – Russell H. Morgan Department of Radiology and Radiological Science, Johns Hopkins University School of Medicine, Baltimore, MD, United States; 9 – F.M. Kirby Research Center for Functional Brain Imaging, Kennedy Krieger Institute, Baltimore, MD, United States; 10 – Department of Electrical Engineering and Computer Sciences, University of California, Berkeley, CA, USA; 11 – Helen Wills Neuroscience Institute, University of California, Berkeley, CA, USA; 12 – School of Electrical Engineering (EIE), Pontificia Universidad Catolica de Valparaiso, Valparaiso, Chile; 13 – High Field MR Centre, Department of Biomedical Imaging and Image-Guided Therapy, Medical University of Vienna, Austria; 14 – Buffalo Neuroimaging Analysis Center, Department of Neurology, Jacobs School of Medicine and Biomedical Sciences at the University at Buffalo, Buffalo, NY, USA; 15 – Center for Biomedical Imaging, Clinical and Translational Science Institute at the University at Buffalo, Buffalo, NY, United States; 16 – Department of Medical Physics and Biomedical Engineering, University College London, London, UK; 17 – MRI Research Institute, Department of Radiology, Weill Cornell Medicine, New York, NY, United States; 18 – Department of Radiology, Mayo Clinic, Jacksonville, FL, United States; 19 – MRI Research Institute, Departments of Radiology and Biomedical Engineering, Cornell University, New York, NY, United States

**Keywords:** Quantitative Susceptibility Mapping, ISMRM Study Group, Clinical Brain Research, Magnetic Resonance Imaging, Data Acquisition, Data Analysis

## Abstract

This article provides recommendations for implementing quantitative susceptibility mapping (QSM) for clinical brain research. It is a consensus of the ISMRM Electro-Magnetic Tissue Properties Study Group. While QSM technical development continues to advance rapidly, the current QSM methods have been demonstrated to be repeatable and reproducible for generating quantitative tissue magnetic susceptibility maps in the brain. However, the many QSM approaches available give rise to the need in the neuroimaging community for guidelines on implementation. This article describes relevant considerations and provides specific implementation recommendations for all steps in QSM data acquisition, processing, analysis, and presentation in scientific publications. We recommend that data be acquired using a monopolar 3D multi-echo GRE sequence, that phase images be saved and exported in DICOM format and unwrapped using an exact unwrapping approach. Multi-echo images should be combined before background removal, and a brain mask created using a brain extraction tool with the incorporation of phase-quality-based masking. Background fields should be removed within the brain mask using a technique based on SHARP or PDF, and the optimization approach to dipole inversion should be employed with a sparsity-based regularization. Susceptibility values should be measured relative to a specified reference, including the common reference region of whole brain as a region of interest in the analysis, and QSM results should be reported with – as a minimum – the acquisition and processing specifications listed in the last section of the article. These recommendations should facilitate clinical QSM research and lead to increased harmonization in data acquisition, analysis, and reporting.

## 1. Introduction

Brain quantitative susceptibility mapping (QSM) has been increasingly performed to identify calcifications and study iron, myelin, and oxygen consumption changes associated with normal brain development or aging and with neurological disease. Diseases of interest include hemorrhagic stroke, multiple sclerosis, Alzheimer’s disease, Parkinson’s disease, amyotrophic lateral sclerosis, and tumors^1–15^. QSM is also increasingly being used in psychiatric disorders such as psychosis^16,17^ or depression^18,19^, where it may reflect neurotransmitter or metabolic imbalances. Changes in brain susceptibility have also been associated with alcohol consumption^20–22^, potentially informing the neural mechanisms through which alcohol affects the brain. Further, QSM has been used for the differentiation between hemorrhages and calcifications^23–26^, anatomical visualization^27–29^ and improved segmentation^30,31^, and for presurgical mapping in deep brain stimulation because it depicts deep gray nuclei targets with exquisite contrast and superior iron source sharpness as compared to other approaches such as T_2_ and T_2_*-weighted imaging^27,32–36^. In conjunction with R_2_ or R_2_* mapping, QSM may also be used to separate diamagnetic myelin and calcification from paramagnetic iron within a voxel^37–46^.

While QSM techniques and their biomedical applications have been extensively reviewed^3,6,7,47–56,57(p31),58,59^, and the QSM research community has held two challenges attempting to identify the best susceptibility calculation algorithm^60–63^, there has been no community consensus or white paper on how to best perform brain QSM in the clinical setting. Some vendors have started implementing research-level QSM pipelines for their systems, nevertheless QSM is not yet a product on most magnetic resonance imaging (MRI) systems. On the other hand, demands for a robust “end-to-end” QSM recipe covering acquisition and processing through to presentation in scientific publications have been growing strongly from the neurological imaging community. For example, the need for a QSM consensus method was highlighted at the group discussion of the February 2022 workshop of the North American Imaging in Multiple Sclerosis (NAIMS) Cooperative, as QSM is particularly useful for depicting the paramagnetic rim of chronic active multiple sclerosis lesions^64–72^. In response to this need, QSM investigators in the Electro-Magnetic Tissue Properties Study Group (EMTP SG) of the International Society for Magnetic Resonance in Medicine (ISMRM) established a QSM Consensus Organization Committee to define the scope of the recommendations and determine the current consensus.

This present article presents the scope of the recommendations, describes the approaches used to obtain consensus, reviews of each step of the QSM reconstruction process, and summarizes its specific consensus recommendations. The technical sections present, first, a general overview of the subject matter in the context of the QSM reconstruction process and then the consensus recommendation. Readers interested only in the consensus recommendations may skip the overview sections and proceed directly to the respective recommendation subsections. Readers interested in the technical details and justification of the recommendations are encouraged to read the overview sections. All consensus recommendations are systematically enumerated to facilitate referencing of the individual recommendations in the literature. Where needed, a sub-section with additional considerations is presented. The paper closes with a summary of the recommendations.

### 1.1 Scope

This consensus paper was written as a guide on how to implement QSM in clinical research for clinical researchers not familiar with the technical nuances of QSM. The implementation of the recommendations requires the assistance of technical personnel such as medical physicists or vendors with in-depth knowledge of the MRI scanner and with expertise in image processing and analysis. The main purpose of this paper is to increase the use of QSM in clinical trials and in different patient groups. Guidelines for clinical practice are beyond the scope of the current paper and would require further study.

Recommendations represent a consensus among QSM experts and the community of experienced QSM users at the time of publication. For clarity, the committee decided that the emphasis of the paper would be on 3 tesla (T), the field strength most widely used in clinical brain research, and that general guidance would be provided for other field strengths (1.5 T and 7 T) where possible. Due to the focus on clinical applications, including clinical trials, the recommendations prioritize robustness and simplicity over state-of-the-art acquisitions and processing algorithms, and provide a starting point for application-specific improvements. Areas in which the committee could not yet reach a consensus (e.g., due to insufficient evidence) are indicated as such in the paper.

### 1.2 Organization

**Figure 1:**
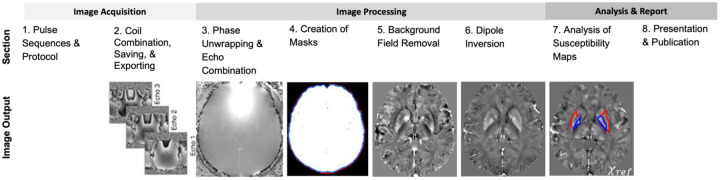
This consensus paper comprises eight sections. The first two sections cover image acquisition: 1) pulse sequences and protocol and 2) coil combination, saving, and exporting. The next four sections cover image processing: 3) phase unwrapping & echo combination, 4) creation of masks, 5) background field removal, and 6) dipole inversion. The last two sections cover analysis and presentation in scientific publications: 7) analysis of susceptibility maps, and 8) presentation and publication. The image output from each section is further detailed in the corresponding section.

The article is organized into eight sections, covering data acquisition, image processing, image analysis, and presentation of QSM studies in scientific publications (Fig.1). Data acquisition is split into two sections: 1) pulse sequences and protocol, and 2) coil combination, saving, and exporting; image processing is split into four sections corresponding to four processing steps: phase unwrapping and echo combination, creation of masks, background field removal, and dipole inversion; the image analysis section focuses on the analysis of susceptibility maps; and the last section on presentation covers reporting in scientific publications. The contributions of the members of the QSM Consensus Organization Committee are listed in the Supplementary Materials I, Section S1.1 along with an overview of the history and approach of the initiative (Section S1.3). The Acknowledgement section lists all individuals who contributed significantly to the consensus recommendations in verbal or written form.

The paper is accompanied by harmonized pulse sequence protocols for several major platforms (current software versions in 2022) as well as sample code to perform the recommended processing steps (see Supplementary Materials II) using the Sepia toolbox^73^ [available at https://github.com/kschan0214/sepia]. In addition, curated online resources are provided and will be updated as the field progresses. This information is detailed in the Supplementary Materials.

### 1.3 Background

Tissue magnetic susceptibility is directly related to tissue chemical composition. A substance gaining a magnetization opposing an externally applied magnetic field, e.g. calcium, is called diamagnetic. Diamagnetism is due to electron orbit perturbation. A substance gaining a magnetization in the same direction as the external field, e.g. iron, is called paramagnetic. Paramagnetism is due to spin alignment of unpaired electrons, which have a magnetic moment 658 times greater than that of the hydrogen nucleus. Most biological tissues, like the human brain, have a susceptibility close to that of water, which is diamagnetic with a value of roughly −9 parts per million (ppm). In the brain, iron deposition makes tissue less diamagnetic than water, and calcium deposition and the presence of myelin make tissue more diamagnetic than water^4,11,15,74–76^.

The static magnetic field of magnetic resonance imaging (MRI) scanners, *B*_0_, affects electrons in tissue and, in linear materials, induces a magnetization proportional to *B*_0_ with the proportionality constant defined as magnetic susceptibility *χ* of the tissue. The induced tissue magnetization generates a field that is nonlocal and extends into the space surrounding the magnetization source, thereby inducing field inhomogeneities. These inhomogeneities can be described mathematically as a convolution of the susceptibility distribution with the unit dipole kernel^77–79^. During MRI signal generation, water proton spins experience this tissue field. In particular, gradient-recalled echo (GRE) imaging is very sensitive to this tissue field. Consequently, there is complex destructive interfering or dephasing by the tissue field variation within a voxel seen as hypointensities or blooming susceptibility artifacts on the GRE magnitude images, which is known as T_2_*-weighted or susceptibility-weighted imaging^80–82^. The GRE phase images, traditionally discarded, represent the tissue field inhomogeneities averaged over the voxel. The process by which tissue magnetic susceptibility is computed from the magnetic field measurement is called quantitative susceptibility mapping (QSM)^83,84^. Several detailed review papers have been published about the technical foundations and clinical applications of QSM, and we refer the interested reader to these articles for a comprehensive overview of the technique^5,13,49,50,52–56,59,85–93^.

## 2. Pulse Sequences and Protocol

This section describes recommendations to robustly acquire data for QSM.

### 2.1. Overview

Most of the major MRI manufacturers, if not all of them, provide a radiofrequency (RF)-spoiled 3D multi-echo GRE pulse sequence from which one can obtain phase images for QSM in addition to the standard T_2_*-weighted magnitude images. The GRE sequence is probably the most elementary sequence in the MRI sequence tree, consisting in its simplest form of an RF excitation pulse followed by acquisition of a gradient recalled echo, which for the multi-echo variant is repeated at various echo times (TEs) before the next excitation pulse is applied. This sequence is often used for calibration steps, such as obtaining a field map for *B*_0_ shimming.

Optimizing a GRE sequence to maximize QSM contrast shares many of the principles for optimizing T_2_*-weighted contrast-to-noise ratio or T_2_* mapping^94^. We recommend the following principles for designing a QSM acquisition protocol:

- Aim to set the last TE equal to at least the T_2_* value of the tissue of interest. For example, if targeting deep gray matter, the T_2_* of the putamen can be a good reference (55, 30 and 16 ms at 1.5, 3 and 7 T, respectively^95^). The first echo time (TE1) should be as short as possible and the spacing between consecutive echoes (ΔTE) should be uniform throughout the echo train.
- Use the minimum repetition time (TR) and set the flip angle to the Ernst angle (*θ*_Ernst_ = cos^−1^⁡(*e*^−TR/T^1)) for the target region. Most deep gray matter structures have a T_1_ close to that of white matter, and white matter represents the largest volume fraction in the brain. Hence, the Ernst angle may be calculated for white matter or deep gray matter with T_1_ = 650, 850 and 1220 ms at 1.5, 3 and 7 T respectively^96^.
- Use three or more monopolar echoes. While QSM can be achieved with one echo and two is the minimum number of echoes needed to separate the intrinsic transmit RF phase from the magnetic field-induced phase (see the Section 3 below), the use of a larger number of echoes (e.g., 5 echoes) will benefit the phase signal-to-noise ratio (SNR) for a range of tissues^97^. As phase SNR is maximal when TE = T_2_*, the use of multiple echoes ensures that high SNR field estimates are obtained for both short apparent T_2_* (venous blood or tissues close to air-tissue interfaces) and other tissues with longer T_2_* values.
- Use the minimum readout bandwidth (BW) that generates acceptable distortions. At 3T, 220 Hz/pixel is often sufficient (two-pixel fat-water shift). Such acquisitions negate the need to use fat suppression for brain applications. Note that as the BW is increased the ratio of the readout duration (1/BW) and echo spacing decreases (as a consequence of the required time to rewind the gradients), which results in a reduction in the SNR of the acquisition. The best trade-off depends on the system’s gradient amplitude and slew rate.
- Use isotropic voxels of at most 1 mm to avoid susceptibility underestimation reported to occur at larger voxel sizes^98^.
- Use 3D acquisition instead of 2D acquisition to avoid potential slice-to-slice phase discontinuities in 2D phase maps that mainly occur in interleaved acquisitions and require additional processing or to avoid slice crosstalk in non-interleaved acquisitions^99^. Note that the 3D spatial coherence of the obtained phase/field maps is of great importance as QSM is ultimately a 3D spatial deconvolution process.
- Use a monopolar gradient readout (fly-back) to avoid geometric mismatch and eddy current related phase problems between even and odd echoes in bipolar acquisitions since these require additional correction in the pipeline used^100^.
- Consider using flow compensation when targeting vessels (e.g., oxygenation studies), but note that flow compensation is often only available and guaranteed for the first echo, while flow artifacts increase in later echoes. Flow compensation also reduces the number of echoes achievable as it increases the minimum TE, assuming a fixed BW. In comparison, flow compensation has a much smaller effect on the resulting QSM values than the choice of QSM processing pipeline^101^.

The following points focus on practical aspects of image orientation, FOV, and acceleration to achieve a whole brain data acquisition in approximately 6 minutes for clinical research on a 3 T system with as few as 8 receive channels using a standard 3D multi-echo RF-spoiled GRE sequence:

- Patients should be scanned in the supine position with the head straight to minimize variability in white matter susceptibility related to myelin magnetic susceptibility anisotropy^102,103^ and microstructural water compartmentation^104^.
- For whole brain acquisition (including cerebellum), it is recommended to use isotropic voxels^98^ and prescribe the imaging volume with the readout direction along the anterior commissure - posterior commissure (AC-PC) line (oblique axial orientation), which reduces the number of phase encoding steps and, consequently, the total scan time^105^. Setting the readout in this direction also restricts eye movement artifacts in the left-right direction, outside of the brain. Alternatively, sagittal acquisitions (readout along the head-foot direction) can be beneficial when the region of interest includes the brain stem but will require larger acceleration factors. The use of tilted imaging slab orientations (oblique axial or oblique sagittal) requires that the data header information (see Section 3 below) specifying the exact slab orientation is supplied to the subsequent processing pipeline, otherwise artifacts will occur in the resulting susceptibility map^106^.
- Parallel imaging and partial k-space coverage can be used to reduce acquisition time, as for any structural MRI acquisition. Below we give general guidelines for GRE brain imaging:
  - If accelerating in only one direction, use acceleration factors <3. If available, non-integer acceleration factors allow fine-tuning the acceleration factors to the available equipment.
  - If available, elliptical k-space sampling (elliptical k-space shutter) and/or partial k-space acquisition (e.g., 7/8) with zero-filling can be used to reduce scan time but note that this can result in a reduction of the effective spatial resolution.
  - If an RF coil with 32 or more channels is available, acceleration in both phase encoding directions by a factor of 2 (2×2) can be used, preferably with CAIPI patterns^107^.
  - If available, similar or higher acceleration factors compared to conventional accelerated imaging may be obtained with incoherent sampling using compressed sensing reconstruction methods^108^ or with deep learning reconstruction methods^109^.

### 2.2. Consensus Recommendation

The recommendation in this section is based on protocols currently available on commercial scanners that do not require a special research agreement with the scanner manufacturer:

2.a. 3D multi-echo RF-spoiled GRE with monopolar readout. Three or more echoes should be acquired and the TE range should include the T_2_* times of the target tissues.
2.b. Minimum TR (given selected TEs).
2.c. Flip angle should be set to the Ernst angle for target tissues (e.g., white matter).
2.d. Whole brain coverage.
2.e. Resolution should be isotropic with a voxel edge length of at most 1 mm non-interpolated at 3T.
2.f. Use accelerated imaging methods (e.g., parallel imaging).
2.g. Use coil arrays with a large number of elements covering the whole brain.

The Supplementary Materials I, Section S1.5 contain sample protocols for 1.5T, 3T, and 7T.

### 2.3. Additional Considerations

Acquisitions for QSM reconstructions can be performed at all clinical field strengths with minimal image distortions using the protocol recommendations provided here, although higher field strengths provide several benefits. Susceptibility contrast and contrast-to-noise ratio (CNR) increase with the static magnetic field once relaxation time changes are considered^110^, and acquisition can be more time efficient at higher fields due to the shorter T_2_*. Our recommended protocols implicitly integrate a compensation for lower SNR at lower *B*_0_ through lower spatial resolution, while keeping the total acquisition times similar. While useful data can be obtained at all field strengths, finer anatomical details in certain applications, such as brain lesions in multiple sclerosis^111^, might be difficult to visualize at 1.5T.

Advanced sequences such as segmented 3D-EPI^112,113^ or Wave-CAIPI^114^ allow drastic decreases in acquisition time but are not available as commercial sequences on all vendors and may require a research agreement with the scanner manufacturer. Nevertheless, it should be noted that some of these methods may have constraints on the number of echoes that can be acquired and 3D-EPI sequences will have increased spatial distortions that may have to be corrected for and that may result in additional artifacts that subsequent processing steps such as background field removal need to account for.

## 3. Coil Combination, Saving, and Exporting

This section recommends effective and practical solutions for generating phase images that can be used for QSM and provides guidance on how to save and convert the format of the phase images in preparation for QSM analysis.

### 3.1. Overview

Modern MRI systems use phased array coils made up typically of 12, 32 or 64 elements, which provide higher SNR than a single birdcage coil and facilitate parallel imaging^115–117^. However, the images from individual coil elements are sensitive to only a part of the FOV and need to be combined to generate a single image – a process that requires consideration of the differences between the coil signals.

**Figure 2:**
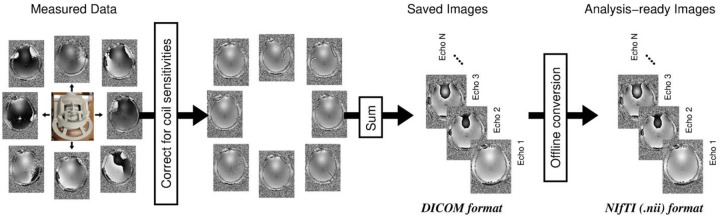
Steps in coil combination, saving, and exporting (illustrated for eight example coils from the 64-channel array). Each of the coils generates a phase image (left), which is modified by the coil sensitivity and other terms which make up the initial phase. The initial phase is removed using methods detailed in the text and referenced publications (center left) and phase images are combined in the manufacturer’s reconstruction and saved for export in DICOM format (center right). QSM analysis software may require the DICOM data to be converted, offline, to NIfTI format (right). Images shown were acquired at 3 T (Siemens Healthineers, Erlangen, Germany; Prisma Fit, VE11C) with a head/neck 64 channel coil and the recommended multi-echo GRE sequence (TE_1_=5.25ms; echo spacing=5.83ms; 5 echoes) using monopolar readout. Additional details are reported in Section 9.1.5. The imaging data and a description of the acquisition protocol may be found in Supplementary Materials II.

**Figure 3:**
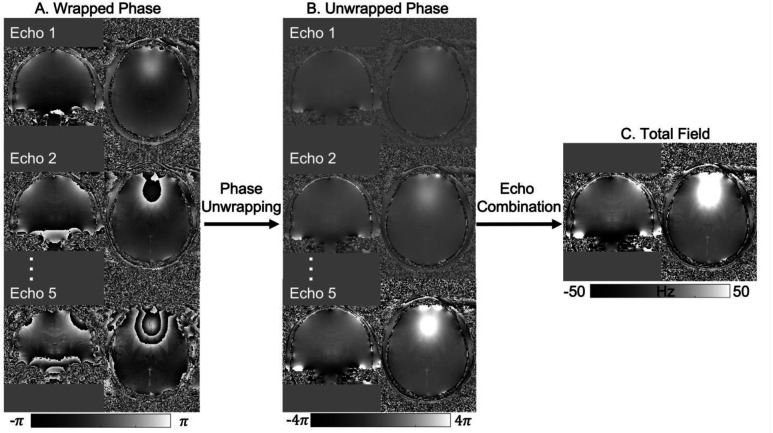
Some scanner manufacturers’ options for processing and saving phase images (like “Sum of Squares”) do not remove coil sensitivities. This may be apparent in the combined phase images having open-ended fringe lines (left). Wraps in phase images combined with the recommended methods are quite symmetric across the brain mid-line (right), and (like contours on a topographic map) either begin and end at the edge of the brain tissues, or form closed loops within the brain.

Neglecting phase wraps (see Section 4), the phase measured with a particular RF coil element *c* in a GRE sequence, *φ*^*c*^, is given by: $\varphi^{c}=\varphi_{\text{B}}(\text{TE})+\varphi_{0}^{c}$. The first part, *φ*_B_(TE), is the phase shift caused by the deviation Δ*B*_0_ of the magnetic field from the uniform main magnetic field, *B*_0_. Neglecting non-linear effects (discussed in Section 9.1.6), *φ*_B_(TE) evolves linearly over time and is the term that is relevant for QSM. The second part, $\varphi_{0}^{c}$, is the phase at TE=0, known as the phase offset (or initial phase) of coil element *c*. This contribution comprises effects that are common to all the RF receive coils in a phased array, such as the phase of the transmit RF field *B*_1_^+^, the effects of tissue conductivity (Liu et al. 2017), gradient delays and eddy current effects, and contributions that are unique to each receive coil, such as the coil sensitivity. Coil-dependent phase offsets must be removed prior to a complex summation of the coil signals, as shown in Figure 2, to avoid destructive interference. Destructive interference leads to reduced SNR and unphysical phase wraps in regions of the image, which cause artifacts in QSM^118^. Complete destructive interference is often associated with phase wraps referred to as “open-ended fringe lines” or “phase singularities” (see Figure 3, left). These ill-behaved wraps do not represent isophase contours and cannot be fully removed by unwrapping (see Section 3 below).

All major 3T MR system vendors have effective solutions for generating phase images from RF array coils on their current software platforms. Most of these techniques remove individual coil sensitivities, which are estimated by referencing to a coil with a relatively homogenous sensitivity over the object (usually the body coil). The reference data is acquired in a separate, fast, automated measurement, and the coil sensitivity correction is carried out on complex data either in k-space^115^ or image space^119^ before extraction of the phase. To combine the signals from each coil element, we recommend using the available ‘on-console’ vendor solutions listed at the end of this section. These methods may not be available on systems running older software versions, in which case phase images can be reconstructed from ‘raw’ (k-space) data offline or, alternatively, phase images can be saved and exported separately for each coil element (e.g., as DICOM-format image data) to allow an appropriate coil combination method to be applied offline. These offline solutions may require additional efforts in 1) dealing with the export and transfer of very large files or a large number of files, which can be problematic in a clinical setting and 2) obtaining research agreements with vendors for proprietary information on the raw data format and on special routines for performing the image reconstruction offline. Several offline possibilities are nonetheless described in the Supplementary Materials III.

A survey of the members of the committee, conducted as part of this study, showed that most respondents use the coil combination methods listed in Section 3.2. A small proportion of users of Siemens systems, particularly with those running older software or those imaging at 7T, use ASPIRE^120^ or the separate channel coil export with an offline solution. In the broader community, many groups continue to use offline solutions for consistency with 7T (where no body coil-based solution exists) or to provide consistency with older studies.

For all systems, data should be exported in DICOM format. However, most QSM tools require data to be in NIfTI format, and a number of tools are available to perform the necessary conversion. We recommend DCM2NIIX (https://github.com/rordenlab/dcm2niix), which is a well-maintained open-source software with compiled versions for macOS, Linux and Windows. It has been tested on data from a range of scanners^121^, including the example data provided with this project. The following alternative programs fulfill a similar function: DICM2NII (https://github.com/xiangruili/dicm2nii), MRIConvert (https://lcni.uoregon.edu/downloads/mriconvert/mriconvert-and-mcverter), and Dicomifier (https://github.com/lamyj/dicomifier). In our survey, DCM2NIIX was the most popular conversion tool, but with a significant spread across the others listed. Format conversion with DCM2NIIX and the analysis steps recommended in this paper preserve the correct image orientation. It should be noted, though, that for non-vendor imaging sequences, some conversion tools and non-standard processing steps (or some combination of these) may not save or handle header parameters correctly. If any of these are used, the researcher should check that left-right flips have not been introduced into images (see Section 8.1.1).

If manufacturers’ product susceptibility weighted imaging (SWI) sequences^122–124^ are used for QSM acquisition, it should be noted that many of these do not allow direct saving or export of unfiltered phase images. Some do allow saving of the processed (e.g., homodyne filtered) phase data, but these are unsuitable for QSM. If raw k-space data are available from SWI scans, it may be possible to generate phase images usable for QSM from these (see Supplementary Materials III). Where available, we recommend that clinical researchers use scanners and sequences which allow saving and exporting of the original unprocessed phase wherever possible, as this greatly facilitates (retrospective) clinical QSM studies and accelerates clinical translation of QSM.

It is important to make sure that phase images are scaled correctly and converted to the correct data type for subsequent analysis. The analysis pipeline supplied with this paper works with a range of data types and arbitrary phase scaling, but some phase unwrapping algorithms and the nonlinear complex fitting algorithm described in Section 4 require phase to be saved as floating-point numbers scaled between −π and π, and this rescaling and data type conversion needs to be performed by the user. Further, note that the sign convention is different for phase values on different vendor systems^125^ and this needs to be checked and corrected where necessary so that relatively paramagnetic tissues (e.g., iron-rich deep-brain regions) have positive susceptibility values in the resulting susceptibility maps.

### 3.2. Consensus Recommendations

We provide recommendations for combining phase images from array coils and saving phase data for each of the major manufacturers, listing the software versions for which these solutions have been tested and whether a research agreement is required. Detailed step-by-step descriptions and solutions for older systems are provided in Supplementary Materials III.

3.a The recommended solution for saving phase images are, for
  - Canon: SPEEDER, a version of SENSE which is available from MPower version 2.3 onwards and allows phase images to be reconstructed through a vendor-provided service password.
  - GE: ASSET, a SENSE-similar solution which reconstructs magnitude, phase, real, and imaginary images without a research key on platforms MR30 onward.
  - Philips: SENSE, which provides well-combined phase images^116^ without the need for a research key from software version 5 onwards.
  - Siemens: “Adaptive-combined with prescan normalize”^126^, which is available from software version VE11 onward in the product GRE sequence.
  - United Imaging: an inter-coil referencing and weighted correction approach which is available from software version v9 without the need for a research key.
3.b Exporting data: Data should be exported in (classic) DICOM format.
3.c Format conversion: if the analysis pipeline requires NIfTI data, DICOM data should be converted to NIfTI using DCM2NIIX.

### 3.3. Additional Considerations

## 4. Phase Unwrapping and Echo Combination

This section describes the methods used to resolve phase aliasing and calculate a field map from multi-echo GRE data.

### 4.1. Overview

**Figure 4.**
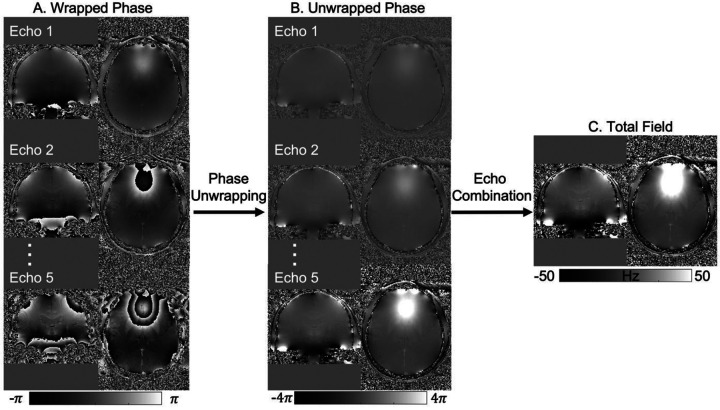
A) Example wrapped phase images at different echo times after proper coil combination (same images as shown on the right-hand side of Figure 2). More phase wraps can be observed at a later echo (bottom). B) Example unwrapped phase images (using ROMEO template phase unwrapping with MCPC3D-S phase offset correction). C) Total field estimation after echo combination using weighted echo averaging.

MRI phase measurements are constrained to an interval of 2*π* and are, therefore, subject to phase wraps or phase aliasing artifacts, i.e., the measured phase *φ* = (*φ*_B_(TE) + *φ*_0_) mod 2*π*. Such phase wraps introduce a phase difference of an integer multiple of 2*π* between the measured phase, *φ*, and the true phase *φ*_B_(TE) + *φ*_0_. Phase wraps are usually visible as discontinuous phase jumps in the phase images (Figs. 3 and 4). To a first-order approximation, *φ*_B_(TE) = 2*πγ*TE ∙ Δ*B*_0_, where γ is the proton gyromagnetic ratio. To obtain an accurate estimate of the field shift, Δ*B*_0_, for QSM, both phase wraps and the phase offset *φ*_0_ need to be removed from the measured phase, *φ*^54^. The coil combination methods recommended in the previous section remove the coil-specific contributions to the phase offset, *φ*_0_, but leave other non-B_0_-related contributions in *φ*_0_, common to all coils, which should be removed by the QSM processing pipeline. Note that the background field removal step (see Section 6 below) removes only harmonic fields (those which satisfy Laplace’s equation) within the brain region and cannot completely remove *φ*_0_ as it contains both harmonic and non-harmonic components^55^(Schweser et al., 2017). Therefore, *φ*_0_ must be explicitly removed for accurate QSM^127^.

Over the years, different phase unwrapping methods have been adapted, refined and applied to MR phase imaging; including time-domain unwrapping methods (with multi-echo acquisition) such as CAMPUS^128^ and UMPIRE^129^, and spatial-domain unwrapping methods such as region-based PRELUDE^130^, SEGUE^131^, and SPUN^132^, path-based best-path unwrapping^133^ and ROMEO^134^, and Laplacian unwrapping^135^. The Laplacian unwrapping method is robust and gives wrap-free phase results even with low SNR but can result in high-frequency errors that propagate into susceptibility maps that are hard to detect visually^136^. It is also noted that Laplacian unwrapping only gives an approximation of the underlying unwrapped phase, especially when using the commonly used Fourier-based implementation, while region-based and path-based unwrapping give quantitatively more accurate estimates of the unwrapped phase^54,134^. Region-based and path-based methods are termed “exact unwrapping methods” below as they give the exact value of the unwrapped phase^134^. When comparing exact unwrapping methods to Laplacian unwrapping, unwrapping errors (e.g., in veins and hemorrhages) are observed to be smaller in the former, improving QSM quantification accuracy, e.g., for oxygenation estimation^137,138^. Therefore, we recommend using exact unwrapping methods.

Multi-echo phase images, e.g., acquired using the recommended protocol (see Section 2), can be combined to achieve a more accurate estimate of the underlying field shift, Δ*B*_0_, than can be obtained from single-echo phase images^97^. This is because combining multi-echo phase images can remove the phase offset contribution and give higher SNR in the estimated tissue field and susceptibility maps (Fig. 4). The optimum approach may depend on the application, but two echo combination methods have been widely used for QSM – nonlinear complex data fitting^139^ and weighted echo averaging^94^.

The nonlinear complex data fitting approach takes into account the Gaussian noise in the complex images^139^ and estimates the field shift, Δ*B*_0_, and phase offset, *φ*_0_, together as parameters from fitting the complex MR signal over multiple echoes, with the requirement of having acquired three or more echoes^139^. This approach usually needs spatial phase unwrapping to be performed after the fitting, i.e., on a scaled field-shift estimate, e.g., 2*πγ* ∙ *δTE* ∙ *ΔB*_0_, with *δTE* being the echo spacing, wrapped again between −*π* and +*π*, as it resolves phase wraps in the temporal dimension. Nonlinear complex data fitting is more robust than linear phase fitting against phase noise at long TEs and around large susceptibility sources, e.g., veins and hemorrhages.

If echoes are acquired over a useful range of TE (depending on T_2_* values of the tissues of interest, see Section 2), the weighted echo averaging approach gives higher SNR for estimating the field shift, *ΔB*_0_, than the complex data fitting approach, which estimates multiple parameters^94,140,141^. Unlike nonlinear complex data fitting, this approach needs the phase data at each TE to be unwrapped first. It also requires explicit removal of the phase offset through subtraction of the estimated phase offset from the phase measured at each TE (for example as in MCPC-3D-S and ASPIRE, where the phase offset can be estimated by extrapolating the linear phase evolution to zero echo time)^120^. Unwrapping errors at longer TEs in voxels with large field shifts and more pronounced noise can be reduced by the “template” unwrapping approach used in ROMEO, which performs path-based spatial unwrapping on an early echo and unwraps other echoes on the basis of the expected linear phase evolution^134^. This, combined with weighted echo averaging, reduces the effect of such errors in the estimated field map.

To improve QSM quality, the spatial noise map generated from non-linear complex fitting^139^, or the phase “quality map” calculated in path-based unwrapping^134^ can be used to mask out voxels with unreliable phase (see Section 5).

Most echo combination methods assume linear phase evolution over TE, ignoring non-linear phase evolution due to microstructure-related compartmentalization effects or biased sampling of the sub-voxel field perturbation (see Section 9.1.6), flow, or signal dropout^142^. Advanced modeling of the phase evolution over time may provide further information about tissue composition and microstructure^37,104,143–148^, but is beyond the scope of this paper.

### 4.2. Consensus Recommendations

4.a. Use an exact phase unwrapping method.
4.b. Perform echo combination before background field removal.
4.c. The optimal pipeline for phase unwrapping and echo combination depends on the acquisition and application. We recommend using either nonlinear complex data fitting followed by spatial phase unwrapping, or weighted echo averaging after template phase unwrapping and explicit phase offset removal.

### 4.3. Additional Considerations

## 5. Creation of masks

This section provides recommendations on creating masks for background field removal (Section 6), dipole inversion (Section 7), and visualization (Section 9).

### 5.1. Overview

Masking is often overlooked when describing a QSM pipeline but is a crucial step^149^, particularly for background field removal (see Section 6). Masking refers to selecting a region of interest (ROI) within the whole field of view and applying a process or function only within this ROI. In QSM, field maps, Δ*B*_0_, are masked primarily because most background field removal algorithms require a mask. In general, masks should cover the largest ROI possible to prevent exclusion of brain tissue with a sufficient signal-to-noise ratio to have reliable phase/field values. This is of special concern for studies of the cortex and the brainstem near the brain border or air-tissue interfaces. Unreliable field map data is composed mostly of extremely noisy voxels resulting from phase noise in regions with very low MRI signal or rapid signal decay. The noise distribution in phase images (and hence, field maps) is generally non-Gaussian and depends on the local magnitude of the signal^150^. In practice, regions with very low MRI signal yield phase noise uniformly distributed throughout the whole −π to π range, obscuring any underlying phase contrast information^150^.

Masking is a binary operation. Voxels with mask values of 1 (or Boolean “true”) are included in the selected ROI and voxels with mask values of zero (or Boolean “false”) are excluded. Masks may be created by using heuristic thresholding operations on available subject images, including magnitude images, T_2_* maps, quality maps, or SNR maps. Masks created from differently thresholded images may also be joined or combined to exclude or include regions based on different criteria. In addition, segmentation algorithms may be based on pre-learned shapes or on the optimization of functionals^151–157^. In particular, the Brain Extraction Tool (BET)^158^ from the FMRIB Software Library (FSL) is a widely-used method for brain masking (skull stripping), although it may fail when pathologies or injuries are present^159,160^. BET is a magnitude-image based algorithm that effectively removes non-brain tissues, air, and bone from magnitude images of the head. When acquiring multiple echoes, using the last-echo magnitude image for BET masking is robust to remove regions with rapid signal dropout^161^, which is undesirable if such regions are of interest. A more balanced approach with a larger ROI selection is achieved by using magnitude images combined across TEs (e.g., using sum of squares or weighted averaging). Alternatively, the magnitude image of the first echo can be used for brain extraction, with the use of a phase-quality map to further exclude voxels with unreliable phase values, as described below. Alternatives to BET include standard template-based brain-extraction^162^. Deep learning segmentation alternatives may also be considered, as this is a rapidly developing field^163–166^.

QSM is also vulnerable to errors and artifacts arising from unreliable phase data that may not be directly reflected in the corresponding magnitude data. These may be caused by coil combination errors, flow in vessels, and other factors. For this reason, it has been proposed to use phase-based quality maps in addition to magnitude-based masking to refine masking^161^. A straightforward method to obtain a phase quality map is to threshold the inverse of the noise map provided by the complex nonlinear multi-echo fitting algorithm (described in Section 4) at its mean value^139,161^. The thresholding at the mean value maintains an adequate number of voxels, as it is applied to the entire field of view (FOV), and the distribution of values exhibits bimodal characteristics. This approach effectively distinguishes between reliable and unreliable voxels, serving as a suitable initial approximation. However, modifying the threshold factor (e.g., by multiplying it by 1.2) may yield further enhancement in the results. Some exact phase unwrapping algorithms provide an alternative source of phase-based quality maps^134,167^. Setting a threshold for phase-based quality maps can help to identify voxels within the brain with unreliable phase values, and to provide a better estimation of the brain boundary^149,161,168^.

Most phase unwrapping and echo combination algorithms do not require masking^54^ but suppressing extraneous data by masking can speed up some algorithms and improve their robustness. In contrast, almost all background field removal algorithms require masking to define the region of interest, outside which the susceptibility sources are classified as background field sources (see Section 6)^55^. Notable exceptions (i.e., background field methods that do not require masking) are recent deep learning approaches such as SHARQnet^169^ and Total Field Inversion (which, however, requires a mask-like preconditioner)^170^. The performance of background field removal algorithms depends strongly on the mask and poor background field removal can negatively affect the quality of the reconstructed susceptibility maps^171,172^. Many dipole inversion algorithms use masks to exclude voxels with unreliable field values from the susceptibility computation or use masks for regularization^83,139,173^. Finally, susceptibility maps should be masked for display purposes to exclude streaks and spurious information outside the brain (see Section 7 on dipole inversion and Section 9 on presentation and publication).

Background field removal methods remove fields induced by all susceptibility sources outside the supplied brain mask. The accuracy of background field removal is lowest at the boundary of the ROI, such as the brain surface, and improves with increasing distance from the brain surface^55^. Although further erosion of the mask after BFR is not explicitly required, it may be employed in specific cases to eliminate residual artifacts at the boundary. Also, many background removal algorithms are not able to recover a reliable local field over the whole ROI mask^55^, and further erosion is unavoidable. It should be noted that some background field removal algorithms (e.g., V-SHARP; see Section 6 below) will result in erosion of the brain mask and the resulting eroded mask should then be used in all subsequent operations and for display.

Holes inside the brain mask lead to the elimination of field contributions from the susceptibility sources within the holes during background field removal. Such holes can occur when thresholded (e.g., phase-based quality) maps are used to refine masking. For example, if a pathology (such as a hemorrhage or calcifications) creates unreliable phase data inside the brain, the affected region could be set to zero in the brain mask. Removing the field perturbations from susceptibility sources within the holes during the background elimination step will render these sources undetectable in the final susceptibility maps, which can be a significant problem in the clinical setting. Therefore, it is important that holes within the brain mask are filled before the mask is used for background field removal. Holes can be reintroduced in the mask used for dipole inversion as an effective way to prevent streaking artifacts from regions with unreliable phase values. This procedure has been included in some algorithms^139,174^. Since dipole inversion is a nonlocal operation, correct susceptibility values may be inferred inside relatively small holes excluded from the dipole inversion mask. To avoid streaking artifacts created by high contrast sources and pathologies, while preserving accurate susceptibility values inside the holes, some recent two-step approaches suggest performing reconstructions with and without holes and then merging both results^138,149,175,176^. This is useful to improve the accuracy of the reconstructions, and to characterize hemorrhages or calcifications.

Although some recent deep learning-based single-step QSM approaches have shown that explicit masking could be avoided^169,172,177–179^, these methods require further study and validation to be considered for clinical applications.

### 5.2. Consensus Recommendations

**Figure 5:**
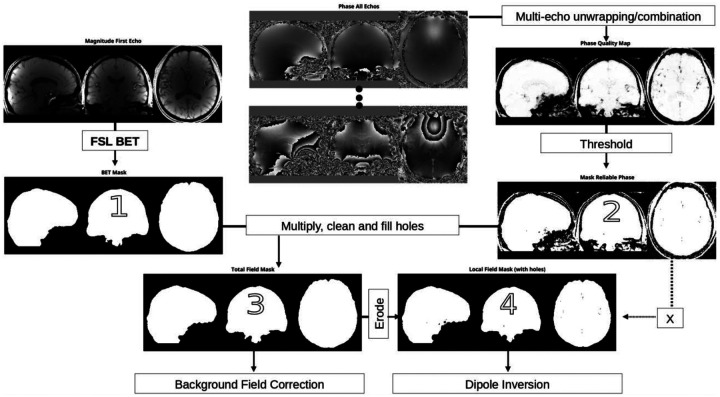
Block diagram of the masking stages. 1) Create an initial mask using FSL BET (Mask 1). 2) Threshold a phase-based quality map to create a reliable phase mask of reliable phase values (Mask 2). 3) Multiply Mask 1 with Mask 2 and fill holes for background field removal. 4) Erode by one or two voxels according to the output of the background field removal algorithm (and optionally reintroduce holes) for dipole inversion. Use Mask 4 without holes filled in for display and reporting susceptibility values. The magnitude and phase images shown are the same as those in Figure 4.

**Figure 6:**
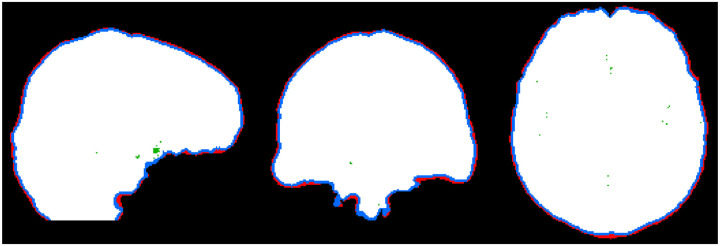
By using the mask or reliable phase (Mask 2), the initial BET mask (Mask 1) can be further improved by removing unreliable phase data near the boundary (red). Mask 3 is used for background field removal (BFR). After BFR, Mask 3 may need to be further eroded depending on the output of the background field removal algorithms (eroded region shown in blue). This is used for visualization of the results and reporting. Unreliable phase data inside the brain can also be masked out for dipole inversion (holes in green, with the final Mask 4 used for dipole inversion in white).

The recommendations below are summarized in Figure 5, and differences between masks (Masks 1–4 in Fig. 5) are highlighted in Figure 6.

5.a. Create an initial brain mask (Mask 1) by applying a whole-brain segmentation tool (such as BET) to either the combined (sum of squares) or the first echo magnitude image. The goal of this initial mask is to remove air, skull and other tissues, while preserving cortical areas. Further refinement is performed in the following steps.
5.a. Create a mask of reliable phase values (Mask 2) by thresholding the phase quality map generated by the multi-echo combination method in Section 4. Multiply Mask 1 with Mask 2.
5.b. After multiplication, holes should be filled to obtain the mask to be used as an input to background field removal algorithms (Mask 3).
5.c. Holes from Mask 2 can be reintroduced to avoid streaking artifacts from unreliable phase data within the brain. For increased accuracy of susceptibility values inside pathological regions of low signal, e.g., hemorrhages and calcifications, mixing data from reconstructions with and without the holes can be performed.
5.d. The calculated susceptibility map should be multiplied by the mask used for background field removal (without holes; Mask 3) before display, reporting of susceptibility values or further analysis.

### 5.3. Additional Considerations

## 6. Background Field Removal

This section provides recommendations for the background field removal step.

### 6.1. Overview

**Figure 7.**
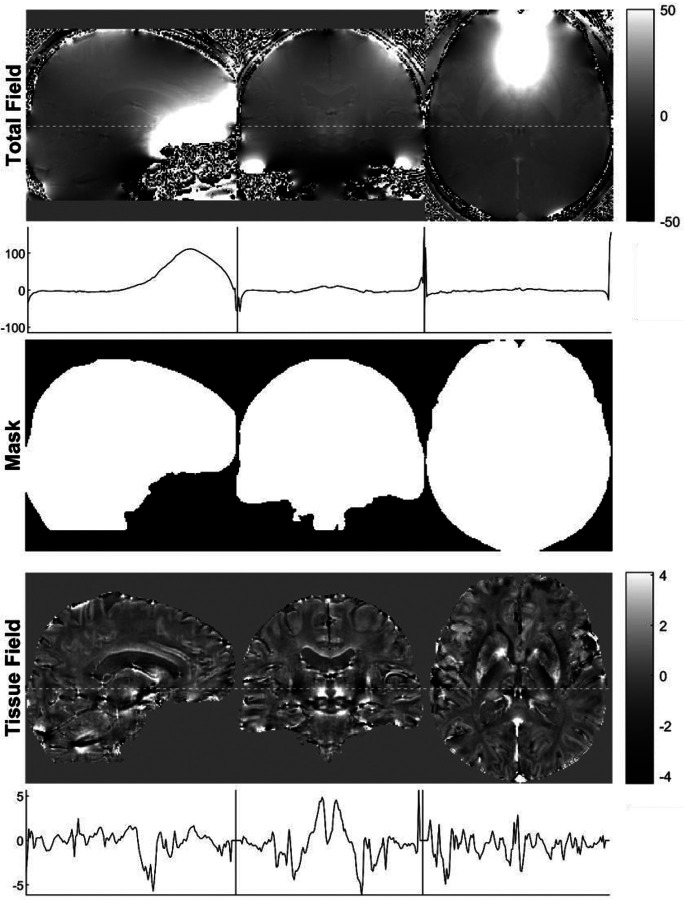
Process of background field removal estimates the background field component of the total field (first row; same images as shown on the right-hand side of Figure 4; unit is Hz) relative to a chosen region of interest (brain mask, third row) and subtracts it from the total field, resulting in the tissue field (fourth row; unit is Hz). The tissue field encodes the spatially varying susceptibility within the brain but is much smaller than the background field. This is illustrated by showing cross-sections (indicated by the dotted lines in the field images) in the total field (second row) and the tissue field (last row). The background field was calculated using the V-SHARP method.

In QSM, the background field is defined as the field generated by susceptibility sources outside a chosen ROI^55^, in our case the brain mask (see previous section). In the brain, the background fields are generated by the tissue and air surrounding the brain. The susceptibility difference between brain tissue and air is approximately 9 ppm^76^, which is almost two orders of magnitude larger than the naturally occurring susceptibility differences within the brain parenchyma. Therefore, background fields can be significantly larger than the tissue field in the brain, but not always are. Certain pathologies, such as hemorrhages, can create a tissue field that is similar in magnitude locally. The term *local field* is also often used in the literature for fields generated by tissue within the ROI, but since the field is a nonlocal property, we use the term *tissue field* here. Removal of the background field from the field map, Δ*B*_0_, allows focusing the inversion (see Section 7) on the spatial susceptibility variations located inside the ROI, which generate the so-called tissue field Δ*B*_t_ (Figure 7). When background fields are not completely removed from Δ*B*_0_, most dipole inversion methods will result in shadowing artifacts and/or experience a slow convergence rate.

Because of the spatial smoothness of the background field, spatial high-pass filtering has been a popular method to suppress background fields in the past. However, high-pass filtering also removes the low spatial frequency component, a major signal component, of the tissue field, which is not acceptable to QSM that requires quantitative accuracy of the corrected field maps. Newer methods that directly exploit the harmonic function property of background fields have replaced heuristic filtering methods^55^. From Maxwell’s equations, it can be derived that the background field is a harmonic field, i.e., it satisfies the Laplace equation within the ROI. A harmonic field is completely determined when it is known on the region boundary. In other words, the solution of the Laplace equation in a region with a given boundary condition is unique^55,180^.

The SHARP (Sophisticated Harmonic Artifact Reduction for Phase data) method^181^ and variants thereof use the spherical mean value property of harmonic functions. This property implies that the average of a harmonic function over an arbitrary sphere centered at any location that fits within the region of interest is equal to the value of the harmonic function at that location. In practice, a radius is chosen that is somewhat large compared to the voxel size to overcome discretization effects. This means that the tissue field can only be computed for voxels that are at a distance equal to the chosen radius away from the boundary of the ROI. This limitation leads to an erosion of the region in which susceptibility can be computed (see also previous section). The most common variant of this method is V-SHARP^182^, which involves multiple partial applications of SHARP with different radii to mitigate the practical implications of the erosion. V-SHARP yields background-corrected field values in the close vicinity of the ROI boundary but the values are not entirely accurate^55^. E-SHARP^183^ and other variants of SHARP^184,185^ overcome the remaining erosion of one voxel required for V-SHARP. Other variants like HARPERELLA^186^ combine SHARP with phase unwrapping. In general, SHARP-based methods perform less well at the boundary of the region of interest^55^. In addition, SHARP-based methods include implicit low pass filtering due to the regularized deconvolution inherent in SHARP, which by itself removes slowly varying components^187^.

The PDF (Projection onto Dipole Fields) method^188^ finds an effective susceptibility distribution outside the region of interest that mimics the field inside that region. It uses the fact that the field generated by those outside sources are approximately orthogonal to those generated by local sources, allowing background field removal to be formulated as a noise weighted linear least squares problem. Because the orthogonality breaks down at the boundary of the region of interest, like SHARP, this method performs less well at the boundary^55,188^.

The LBV (Laplacian Boundary Value) method^180^ assumes that the field at the very boundary of the region of interest is entirely background field and determines a harmonic function that satisfies this boundary condition. An efficient algorithm has been introduced to solve this Laplacian boundary problem^180^. However, because LBV entirely relies on the field estimate at the mask boundary, it is sensitive to phase SNR at the boundary, rendering accurate masking particularly important. Often a small mask erosion is applied to remove low SNR voxels at the boundary. While LBV can perform better than PDF and SHARP in some situations^55^, its performance has been observed to be highly dependent on the mask and on the quality of the field estimates at the mask boundary^189^.

Residual background fields can be suppressed by combining methods, such as applying additional polynomial fitting or V-SHARP after LBV. V-SHARP and PDF typically do not require additional polynomial fitting. B_1_^+^ related contributions in the field map, Δ*B*_0_ (see Section 4 above) are not removed by background field removal methods, but these are avoided when using multi-echo data combined with field fitting as recommended above.

Susceptibility maps are dimensionless and are conventionally calculated and displayed in parts per million (ppm) (see Section 9.1.4). Assuming that the wrapped input phase was correctly scaled to (−π to π) radians, the corresponding scaling can be done either before (on the tissue field map) or after (on the dipole inversion output) using the scale factor: $\Delta B_{t}(\text{ppm})=\frac{\Delta B_{t}(\text{radians})\cdot 10^{6}}{\gamma (\text{radians}\cdot {\text{T}}^{-1}\cdot {\text{s}}^{-1})\cdot B_{0}(\text{T})\cdot \Delta TE(\text{s})}$. When scaling is performed after, care has to be taken to adjust the default regularization parameter when using a total variation based dipole inversion method, as the regularization term scales linearly.

### 6.2. Consensus Recommendations

6.a. Use V-SHARP to achieve good results in many situations, as it is less sensitive to imperfections in brain masking. This comes at a cost of a one-voxel erosion of the brain mask used for dipole inversion (Mask 4 in Fig. 5) at the brain surface and reduced accuracy at the edge of the brain.
6.b. When whole brain mapping (including the cortex and superficial veins) is desired, use PDF. This method will be slightly more accurate throughout the brain. PDF requires a good brain mask.
6.c. Depending on the application, tissue field quality, i.e., the phase SNR especially near the boundary, must be balanced against mask erosion.

### 6.3. Additional Considerations

Single step^112,170,190–192^ or total field inversion methods fit the susceptibility directly to the field map, Δ*B*_0_, or even wrapped phase images. These are currently popular for applications of QSM outside the brain but are still under ongoing development to ensure robustness. Residual background field has been tackled for dipole inversion using weak harmonics modeling^136^. Finally, deep learning^52^ has found application in background field removal as well and is the subject of ongoing development.

## 7. Dipole Inversion

**Figure 8.**
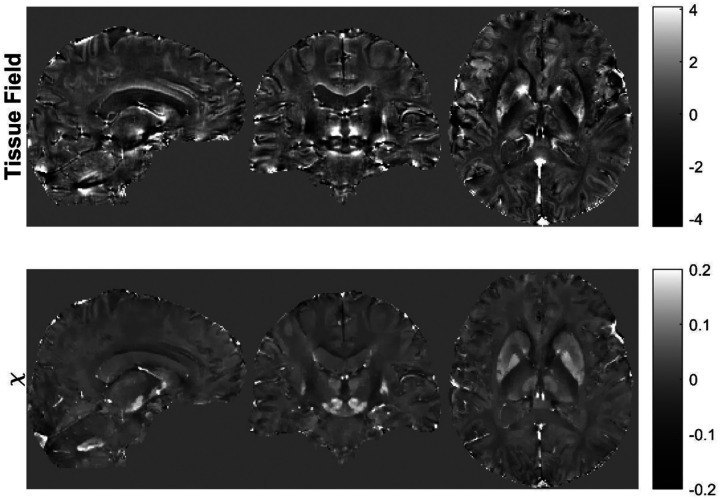
The process of dipole field inversion starts from the tissue field (first row, same images as shown in the bottom row of Figure 7; unit is Hz) and estimates the susceptibility map (second row; unit is ppm).

This section provides recommendations for the field-to-susceptibility inversion step (Fig. 8), which derives from the tissue field map, Δ*B*_*t*_ (with background fields removed; see previous section), a map that is tissue magnetic susceptibility *χ*(*r*) (up to a reference value^193,194^, see Section 8).

### 7.1 Overview

While susceptibility, *χ*(***r***), is a local tissue property, the field is a summation of weighted contributions from the distribution of magnetic susceptibility in all space. Mathematically, this summation can be described as a convolution (*) of the susceptibility with the unit dipole kernel $d(r)=\frac{1}{4\pi }\frac{3cos^{2}\theta -1}{r^{3}}$^77–79^:

[1] ΔB(r)=d(r)*χ(r).

Convolution corresponds to multiplication in the spatial frequency domain, which facilitates its fast calculation and is used in most QSM implementations^77–79^ to accelerate computations. The inversion step performs a deconvolution using the dipole kernel *d(****r***), which reveals the local tissue susceptibility within the region of interest, *χ*_*t*_(***r***), from the background-corrected tissue field, Δ(***r***): Δ*B*_*t*_(***r***) *^−1^ *d*(***r***) = *χ*_*t*_(***r***). However, the dipole kernel value is zero at and very small near the cone surface of the magic angle (54. 7^0^) relative to the direction of the main magnetic field, making this deconvolution a poorly conditioned inverse problem^75,84,195–197^. The measured tissue field, Δ*B*_*t*_, contains deviations from perfect dipole patterns, particularly in regions with small magnitude signal due to lack of water protons (field detectors) or rapid signal decay (largely caused by inhomogeneous fields). While the regions with most extreme deviations are usually eliminated through masking (see Section 5 above), remaining dipole deviations in the estimated field can cause deconvolution errors in the calculated susceptibility map reconstruction, manifesting as streaking and shadowing artifacts^53,198^. Additional information about the unknown susceptibility map, *χ*(***r***), can be incorporated into the solution through regularization to suppress streaking and shadowing artifacts in the solution 25,59,83,84,112,141,173,182,199,200. An optimization approach for incorporating this additional information can be formulated according to Bayesian inference, which is the following minimization problem when approximating the noise in the field as Gaussian:

[2] $$\chi (r)={\text{argmin}}_{\chi (r)}{\left\|w(\Delta B_{t}-d*\chi )\right\|}_{2}^{2}+\lambda R(\chi ).$$

Here the first term is the data fidelity term with spatially varying noise weighting *w* and the second term, *R*(*χ*), is the regularization term with *λ* as regularization strength^83,141^. The minimization problem is iteratively solved with the number of iterations determined by the desired convergence level. The optimal regularization strength (*λ*) depends on anatomy, susceptibility contrast, and SNR, and should be optimized to balance artifact suppression and image sharpness in each imaging protocol and application by varying *λ* using, e.g., the L-curve method^201,202^.

Various regularization strategies have been developed for the inverse problem in QSM^25,53,60,83,84,136,141,173,182,192,197,199–201,203–207^. Available QSM software packages include FAst Nonlinear Susceptibility Inversion (FANSI)^173^ (https://gitlab.com/cmilovic/FANSI-toolbox), Morphology Enabled Dipole Inversion (MEDI)^201,208^ (http://pre.weill.cornell.edu/mri/pages/qsm.html), and STI Suite^209^ (https://people.eecs.berkeley.edu/~chunlei.liu/software.html).

Total variation regularization has performed favorably in the two QSM reconstruction challenges^60,63^. Both FANSI and MEDI provide specific implementations of sparsity regularization with openly accessible source code. Specific implementation examples including zero-referencing to the cerebrospinal fluid (CSF) may be included as an extra regularization that provides the CSF-uniformity verification on the QSM output with an additional benefit of further reducing streaking and shadowing artifacts,^193,210^ but are associated with other limitation as discussed in the next section.

The simple sparse regularizer using L1 norm of the gradient (i.e., the total variation, TV) is a standard approach for brain QSM, as exemplified in MEDI, one of the most popular algorithms. The performance of the TV approach for brain QSM in terms of accuracy and robustness was well established in the 2019 QSM Reconstruction Challenge, particularly in the presence of strong susceptibility sources, such as hemorrhages or calcifications, using nonlinear forward signal modeling^60,63^. Recent developments in deep learning based QSM reconstruction represent an exciting avenue for improving QSM performance^52,179,211,212^. However, while there are instances where these methods yield better results than classical methods, the performance of these methods did not reach those of the best classical methods in the QSM challenges potentially due to generalization issues from limited training data^60,63^.

Some algorithms do not incorporate spatial constraints for suppressing streaking and shadowing artifacts but explicitly modify the dipole kernel instead^53,198^, for example, the thresholded k-space division^192,197,213^. Implicit regularizations based on the number of iterations may work, but these methods have limited denoising capabilities and may be less robust than the sparsity regularization optimization approach^59,205,214^.

### 7.2. Consensus Recommendation

7.a. Use an optimization approach for dipole inversion with a sparsity type regularization that is commonly used in compressed sensing^53^. Specific sparsity types include L1-norm, total variation, and generalized total variation, which likely provide similar outcomes. Future algorithm developments and evaluations are needed to provide a more specific consensus on the sparsity type.
7.b. Use the default sparsity type, regularization strength and number of iterations in a QSM software, such as the processing pipelines recommended here (Supplementary Materials II), including FANSI, STI Suite, and MEDI, where these default parameters have been optimized for common brain protocols. If the acquisition protocol recommended here (Supplementary Materials I, Section S1.5) is substantially altered, researchers should perform an L-curve optimization or other method on at least one typical case with the specific study protocol to finetune the regularization strength and iteration number and then fix these parameters for the same protocol.

### 7.3. Additional Considerations

There may be streaking artifacts coming from strong susceptibility sources near borders and within the brain interior region. Major causes include the breakdown of the Gaussian noise assumption and other errors in the determined field^139^. These artifacts may be suppressed using methods such as masking out or reducing the weight of less trustworthy voxels in the optimization^139^. The border streaking can be removed by improving the brain mask^149,215,216^. The interior streaking can be reduced using techniques to improve convergence such as preconditioning^170,217^, and using in-painting techniques to compensate for field errors such as MERIT^139^ and L_1_ data fidelity^174^.

There may be shadowing artifacts coming from residual background fields. This shadowing can be reduced by improving background field removal such as harmonic incompatibility removal^136,218^ and by suppressing slowly varying spatial frequency components through regularization^219^ or preconditioning^53,170^.

## 8. Analysis of Susceptibility Maps

**Figure 9:**
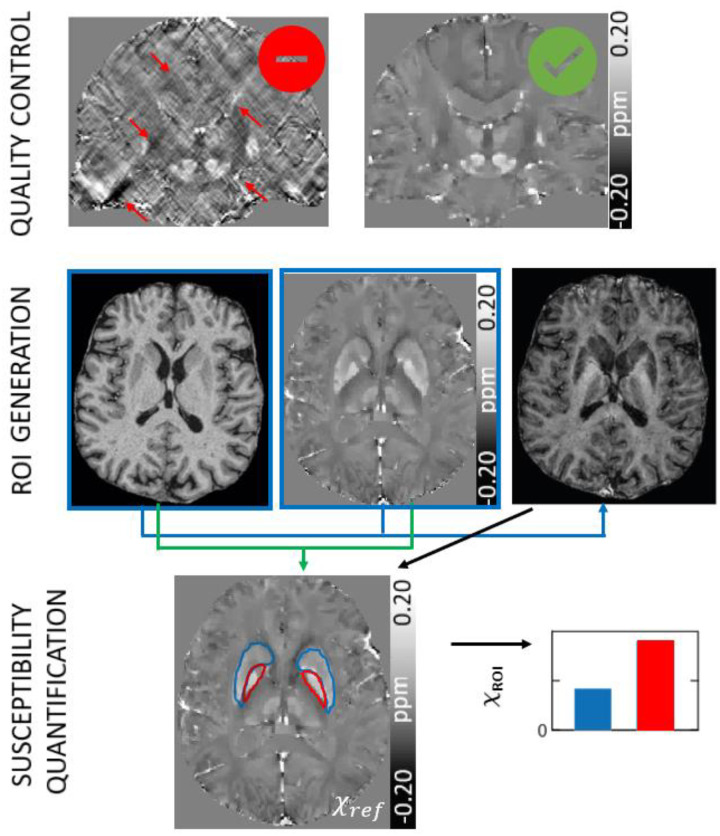
Schematic for susceptibility map analysis in case of a study interested in susceptibility values of the putamen (blue ROI on the susceptibility map, *χ*_*ref*_) and globus pallidus (red ROI). Data with streaking artifacts that affect the ROIs need to be excluded (or recalculated when applicable to all study data). ROI generation benefits from the inclusion of susceptibility contrast, e.g., by calculation of hybrid images (blue) or use of T_1_-weighted and susceptibility data (green). Susceptibility maps need to be referenced, then regional average susceptibility values (*χ*_*ROI*_) can be computed from referenced susceptibility maps (*χ*_*ref*_). The shown susceptibility map without artifacts is the same as the one in Figure 8.

This section provides recommendations for quality control and referencing of susceptibility maps, the quantification of susceptibility values, and the visualization of brain structures on susceptibility maps and derived contrasts in the context of clinical research performing group studies. The physical background for the consensus recommendations is briefly summarized. Possible tools to facilitate susceptibility quantification of brain structures and lesions as well as for group analyses are provided in Supplementary Materials I, Section S1.6. Figure 9 summarizes the recommendations of this section.

### 8.1. Overview

#### 8.1.1. Quality control

Only a few tools for fully automatic QSM calculation and evaluation directly from scanner DICOM data exist to date that perform all steps outlined in Sections 2 to 8^149,220^. Some of these tools may not be suitable for all possible QSM applications due to assumptions on patient cohorts of the implemented mask generation algorithms (see Section 5 above) or due to the need to adjust reconstruction parameters depending on the data (see Section 7 on dipole inversion). Most QSM applications still require multiple processing steps, which can result in error amplification/propagation or inconsistencies between steps, rendering QSM workflows prone to i) reconstruction artifacts (a list of common reconstruction artifacts is provided in Table 1) and ii) calculation errors of region-specific susceptibility values. Particularly, the use of one or several masks to exclude unreliable phase data and for background field removal during QSM calculation can result in missing areas in computed susceptibility maps especially close to air-tissue interfaces. When voxels in those regions are not properly excluded in subsequent analyses of the susceptibility maps (e.g., in ROI-based analyses), regional mean values may be biased by these erroneously included zero-valued voxels in the susceptibility mean value calculations. The issue can be resolved by incorporating the eroded background-correction mask (Mask 4 without holes in Fig. 5) in the ROI masks. Deviation from the radiological orientation (right-left flip) in the final susceptibility maps (see also Section 3.1) can be a potential issue arising from the combination of different toolboxes when using other tools than those recommended here or as a result of erroneous use. These flips can have detrimental consequences in the clinical setting but can be revealed relatively easily comparing brain features between QSM and the original GRE magnitude images on the scanner console, especially when the subject’s head was tilted to the right or left (about the H-F, A-P or both of these axes) for test purposes (see Section 3.1), a step that should always be done if custom pipelines are used.

#### 8.1.2. Referencing and choice of reference region

QSM can only assess relative susceptibility differences between tissues as phase data reflect field distortions due to these underlying spatial susceptibility differences. Susceptibility values are therefore given up to a reference^56,194^. To obtain susceptibility values that are comparable between repeated measurements, subjects, and scanners, consistent referencing of susceptibility maps is required. In QSM, internal reference regions are used. External reference regions are not generally used because it is not currently possible to measure phase differences between disconnected spatial regions separated by noise and perform consistent background field removal for both the brain tissue and the external reference region. The ideal choice of a reference region for brain QSM is still under debate^194^. Different regions used in the literature come with certain advantages and disadvantages and will lead to different susceptibility values in the resulting susceptibility map. For example, assuming an ROI’s average susceptibility value is 0.010 ppm when computed from a susceptibility map referenced to the whole brain (with assumed mean susceptibility of whole brain −0.001 ppm), this ROI susceptibility value will be 0.008 ppm when computed from a susceptibility map referenced to the CSF (assumed mean susceptibility of CSF 0.001 ppm).

In the case of widespread pathology such as in multiple sclerosis or Alzheimer’s disease, there might not be an ideal choice of reference region. Larger reference regions are generally advantageous over small-sized regions, which are more affected by potential local lesions, reconstruction inhomogeneities and other artifacts (less averaging), which are then propagated to all other regions in the map by the referencing process. This issue reduces statistical power and therefore 3D segmentation of reference regions is advisable to include a greater number of voxels. Consequently, whole-brain referencing is considered stable (largest possible mask) and reproducible (whole-brain mask readily available in all reconstruction pipelines).

The dependence of white matter apparent susceptibility on the fiber orientation with respect to the main magnetic field due to the geometry and complex microstructure of white matter fiber bundles^102,104,146,221,222^ (see also Section 9.1.6) can be a source of additional variability when using a reference that includes white matter regions. Another challenge of referencing is that pathology or effects of age alter white and gray matter integrity, specifically myelination and brain iron levels, especially in deep gray nuclei^223–228^. In the case of widespread pathology when no ideal reference region exists, two reference regions could be used to evaluate if the choice of reference region affects the study results. If, for example, whole brain and CSF were used as reference leading to the same significant differences between patients and controls, the results can be assumes with greater confidence to originate from the presence of pathology, instead of being an artifact from susceptibility referencing^229^.

For local pathology, the use of contralateral or surrounding tissues as reference is an effective strategy to avoid introducing artificial susceptibility differences due to using a reference region affected by pathology.

Table 2 lists advantages and disadvantages of common reference regions. More details on referencing can be found in dedicated literature^193,194^. We recommend referencing with regions that are commonly used in the literature. In addition, we recommend that studies report the mean and standard deviation of the susceptibility (after referencing) in other regions that are or have been widely used for referencing along with their hypothesis-driven regions of interest. This approach will promote reproducible research as it facilitates the comparison of susceptibility values between studies and enables post hoc re-referencing for meta-analyses. While no normative susceptibility values exist, literature values^230^ can serve as precedence reference when comparable subjects are studied, e.g. healthy controls of similar age.

#### 8.1.3. Effect of segmentation on susceptibility quantification (iron, white matter changes, lesions, vessels, oxygenation)

An accurate segmentation of ROIs is essential to uncover subtle changes in regional susceptibility values that might indicate pathology, or to establish normative values. While manual segmentation of regions by multiple expert readers is the gold standard for quantification of regional susceptibility values, this strategy is very time-consuming and therefore not feasible in larger studies. Many available automated neuroimaging segmentation tools are optimized for use with T_1_-weighted images or require T_1_-weighted input data^231^. However, when using these methods for the analysis of susceptibility maps, the segmentation and registration accuracy in many structures of interest (e.g., basal ganglia) can depend on T_1_ contrast^232^, which is also affected by tissue iron^233,234^, and the generally low visibility of some deep gray matter regions on T_1_-weighted images^235^ (depending on sequence parameters). Consequently, ROI-based methods that rely solely on T_1_-weighted contrast may be biased and suffer from inaccuracies. Previously, it has been shown that the use of a QSM or hybrid QSM-T_1_-weighted contrasts for template generation improves atlas and voxel-based analyses^30,31^. Therefore, using multi-contrast segmentation can be considered the best approach to avoid template bias^236^. A list of recommended tools can be found in the Supplementary Materials I, Section S1.6. Partial volume effects might strongly affect susceptibility quantification both for voxel-based and ROI-based analyses, especially for small structures with relatively high susceptibility values such as veins^237^. This could be corrected for by eroding of ROIs^101^, only using high susceptibility voxels (in case of positive susceptibility)^238^ or using a partial volume map for correction^239^.

### 8.2. Consensus Recommendations

8.a. When ROIs are affected by artifacts, exclude data by automated detection of outliers or outlier regions, use of image quality measures or visual inspection.
8.b. Ensure that analysis methods do not include voxels of the susceptibility map with unreliable values, e.g., that lie outside of the eroded background field removal mask (see Section 5 above; Mask 4 without holes in Fig. 5).
8.c. Always reference susceptibility maps to an internal reference region before performing further analyses.
8.d. When choosing a reference region, consider the study design, influence of pathology, how pathology could bias the study findings and discuss accordingly. For widespread pathology, cross-checking results using two different reference regions (e.g., whole brain and CSF) can be considered safe to exclude bias.
8.e. Segment reference regions in 3D.
8.f. Always include commonly used reference regions in your analysis and report mean and standard deviation in these regions along with those in other ROIs.
8.g. Consider incorporating QSM contrast in ROI segmentation or ensure that T_1_w-based methods are accurate.

### 8.3. Additional Considerations

## 9. Presentation and Publication

### 9.1. Overview

The purpose of the recommendations in this section is to facilitate the interpretation and replicability of future findings with QSM, future meta-analyses, and the comparison among studies. To this end, the general recommendation is to report as much information as possible regarding:

1. Data acquisition (hardware and scan parameters);
2. Reconstruction pipeline and analysis procedure; and
3. Results.

Depending on the study and on the journal in which the study will be published, the degree of information detail that can be reported may vary. The members of the QSM Consensus Organization Committee asked themselves, through a multiple-choice grid form, whether each information entity relevant for QSM should be reported *always* (*a*) or only *depending* (*d*) on the study and on the journal, or if it is *unnecessary* (*u*) to report the entity. Each item in the poll was assigned a score *S* = (*A* + ⁡0.5*D*)/(*A* + *D* + *U*), where *A*, *D* and *U* are the number of *a*, *d* and *u* responses collected for that item, respectively. The reporting of specific items was considered essential if there was unanimous consensus in reporting them among the authors (*S* = 1). Items that were not considered essential were assigned a “traffic light ranking” (green for 0.75 ≤ *S* ≤ 1, orange for 0.5 ≤ *S* ≤ 0.75, and red for *S* ≤ 0.5). Standardized tables are provided to facilitate the reporting of a broad set of items. For the purpose of availability, unless there is limited space for particular journals, we recommend that items with S>0.5 be reported as other investigators may need this information. It is considered essential to report these items if they vary within the same study (e.g., if different scanners or different software releases are used within the same study). Potential limitations and confounds should always be discussed. The last part of this section reviews some important aspects that should always be considered when interpreting and presenting QSM findings in scientific papers.

#### 9.1.1. Acquisition hardware

Ideally, the acquisition hardware is described in one sentence reporting the scanner field strength, model, vendor, software release version, and type of coil used (including the number of channels). Table 3 provides an overview of consensus recommendations pertaining to acquisition hardware.

#### 9.1.2. Acquisition sequence type and parameters

The QSM Consensus Organization Committee considered it as essential to indicate the acquisition sequence type (e.g. GRE, as recommended in this paper, or EPI; specify if the sequence is 3D or 2D) and several acquisition parameters including number of echoes, TEs, TR, flip angle, bandwidth, resolution and scan duration. Table 4 provides an overview of consensus recommendations pertaining to acquisition sequence type and parameters.

#### 9.1.3. Reconstruction pipeline and analysis

It is considered essential to describe the toolbox and reconstruction pipeline and list the algorithms used. The numerical values of parameters used should be listed, even if they were the default parameters. Table 5 provides an overview of consensus recommendations pertaining to the reconstruction and analysis pipelines.

#### 9.1.4. Displaying figures

When displaying quantitative susceptibility maps, do not use rainbow, jet, or similar types of non-linear colormaps, which introduce the erroneous perception of artificial edges in some parts of the range, hide existing edges in other parts of the range, and lack intuitive perceptual ordering^240–242^. In the absence of a motivation for doing otherwise in particular studies, the use of a linear, perceptually uniform colormap should be preferred; the use of a linear gray-scale map enables consistency with the vast majority of the published literature. This applies also to phase data, to enable a clear representation of phase wraps and/or possible errors such as open-ended fringe lines. Contrast windowing should be adjusted to avoid saturation of relevant brain areas (i.e., completely black or white appearance). A typical window adapted to healthy brain QSM is [−0.2, +0.2] ppm. The windowing should always be reported, by either using an intensity bar or writing the information in the figure caption. Susceptibility maps should be displayed through the eroded mask used for background field removal (see Section 5), to avoid representation of artifactual data outside the brain.

#### 9.1.5. Sample paragraphs

Representative paragraphs describing data acquisition, processing and QSM calculation in a scientific paper are provided in the following. The description refers to the images shown in Figure 8.

“Data were acquired on a 3 T scanner (Prisma Fit, Siemens Healthcare, Erlangen, Germany; VE11B) using the built-in whole-body RF transmit coil and a 64-channel receive-only head/neck coil. The acquisition sequence was a 3D GRE multi-echo with pure axial orientation with the following scanning parameters: TR = 33 ms, 5 monopolar echoes acquired at TE_1_ : ΔTE : TE_5_ = 5.25 : 5.83 : 28.57 ms, flow compensation for the first echo in the readout (AP) and ‘slice’ encoding (HF) direction, FA = 15°, pixel bandwidth = 220 Hz, elliptical k-space shutter, covering a field of view (FOV) of 256(AP)×176(LR)×144(HF) mm^3^ with matrix size=256×176×144, resulting in isotropic voxels of size 1mm^3^, with GRAPPA acceleration factor=2 in the phase encoding (LR) direction. Scan duration was 6 minutes 34 seconds. The full QSM reconstruction was performed in Matlab R2021a (MathWorks, Natick, MA, USA) using the SEPIA toolbox^73^ (v1.2.2.4) for integration of the various processing steps described hereafter. ROMEO total field calculation^134^ (v3.5.6) was used for echo phase combination. Brain masking was obtained with FSL BET^158^ on the first-echo magnitude image, using default settings. V-SHARP^182^ with spherical mean value filtering sizes from12 mm to 1 mm was used for background field removal. Quantitative susceptibility maps were obtained using FANSI^173^ (v3) with gradient L1 penalty of 0.0005 and gradient consistency weight of 0.05. Susceptibility values are expressed in parts per million (ppm) and have been referenced to the average susceptibility in the brain mask (imposed to zero by the adopted processing pipeline).”

A complete description of the QSM calculation pipeline can be found in the Supplementary Materials II.

#### 9.1.6. Interpretation of results

Potential limitations and confounds related to QSM should always be taken into account.

A potential confound that can affect the extraction of quantitative susceptibility values from MRI phase/frequency data arises from the fact that, even for a uniform voxel-averaged susceptibility distribution, the apparent field measured in a voxel depends on the subvoxel distribution and visibility of water protons (the sensors of the MRI signal) around susceptibility perturbers such as iron and myelin. This can lead to a phase shift resulting from the biased sampling of the fields generated by perturbers when sensor and perturber distributions spatially correlate and such correlation is anisotropic^243,244^. An example is water in and around myelinated fibers, whose anisotropic distribution leads to a fiber orientation-dependent shift in apparent frequency which can exceed 10 ppb^104,144,146,221,243,244^. In addition, substantial T_1_, MT, or T_2_* weighting may differentially affect water visibility in the various water compartments and render apparent frequency shifts dependent (in a non-linear manner) on TR or TE^142,143,145^. Especially above 3T, QSM values within and around fibers that run perpendicular to B_0_ should be interpreted with caution. For example, pathological changes in myelin structure but not myelin content in such fiber bundles may lead to QSM changes without actual changes in tissue susceptibility.

Another point of caution with interpretation of QSM are inaccuracies near the edge of the regions selected for the analysis (see Section 5). A notable example are areas near the surface of the brain, where phase data is unreliable (due to, e.g., the prevalence of paramagnetic blood in pial veins), or unavailable (due to the lack of signal in skull), or the tissue phase was partially removed in the background field removal step. Because of this, QSM values in some of cortical grey matter may be incorrect or have reduced spatial contrast and resolution.

Lastly, it should be realized that, when strong regularizations or prior information are used in QSM dipole inversion, potential smoothing and spatial resolution loss may occur or new features may be added^201,245,246^ (see Section 7.1). Some anatomical detail, visible in phase or magnitude GRE data, may therefore be lost in the QSM.

### 9.2. Consensus Recommendations

9.a. Always report at least the essential information regarding the acquisition hardware (Table 3), acquisition sequence type and parameters (Table 4), reconstruction pipeline and analysis (Table 5).
9.b. Representative susceptibility maps and the underlying background-field corrected phase images should be shown in all articles.

To facilitate documenting the reconstruction pipeline, we encourage software developers to enable printing out the values of all relevant parameters in Tables 4 and 5 (including default parameters) and provide suggested descriptions of their toolboxes, which users can re-utilize in their publications.

### 9.3. Additional Considerations

## 10. Summary and Conclusion

This consensus paper has been developed by the QSM Consensus Paper Committee with consideration of suggestions from the whole QSM research community (see Acknowledgements and Supplementary Materials I, Section S1.3). The paper provides recommendations for all steps essential in setting up a successful QSM study in a clinical research setting. The recommendations, intended for a robust but not necessarily state-of-the-art QSM, are based on the current understanding (as of 2023) and should be updated as the QSM field progresses.

In summary, we recommend that data be acquired using a monopolar 3D multi-echo GRE sequence, that phase images be saved and exported in DICOM format and unwrapped using a quantitative approach. Echoes should be combined before background removal, and a brain mask created using a brain extraction tool with the incorporation of phase-quality based masking. Background fields within the brain mask should be removed using a SHARP-based or PDF technique and the optimization approach to dipole inversion should be employed with a sparsity type regularization. Susceptibility values should be measured relative to a specified reference, including the common reference region of whole brain as a region of interest in the analysis, and QSM results should be reported with – as a minimum – the acquisition and processing specifications listed in the final section.

The recommended steps for data acquisition, data preparation and post processing are intended to provide a uniform robust reference starting point for a brain-focused QSM study performed with a clinical scanner. Specialty applications such as the depiction of small structures might require spatial resolutions higher than recommended^247^. In this regard, limitations and further considerations are included in each section, but thorough testing of the processing pipeline is recommended before starting a large patient study.

We hope that the recommendations here will enable many medical research centers to perform comparable QSM studies on scanners from different vendors, and that the standardized acquisition protocols and the processing pipeline provided along with this article will facilitate these studies (see Supplementary Materials I, Section S1.5. and Supplementary Materials II). As more clinical QSM studies are performed, analyzed, and presented in scientific publications, and current and future technical innovations become mature, these QSM recommendations will need to be updated.

## Supplementary Material

1

## Figures and Tables

**Table 1.** Reconstruction artifacts, possible sources, and strategies to identify, mitigate these artifacts and criteria to exclude the data.

Artifact	Streaking and shadowing artifacts 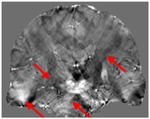	Incorrect susceptibility values 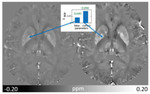	(Regional) strong noise 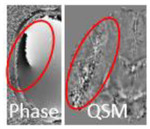
**Typical sources**	large susceptibility differences (air-tissue, calcification, hemorrhage etc.) poor brain mask use of late TEs and dynamic field fluctuations (especially at higher field strength) in combination with unsuitable mask inversion algorithm unsuitable for the data (e.g. presence of strong susceptibility sources) poor choice of reconstruction parameters (for phase unwrapping, background field removal or inversion algorithm) incorrect coil combination	Mismatch between acquired and assumed TE values during the reconstruction, e.g., due to unanticipated acquisition protocol changes	incorrect coil combination suboptimal image acquisition (coverage, 2D)
**Identification**	manual/visual quality control automatic detection of outlier regions on phase images or QSM^220^ (beware of outliers due to pathology) automated histogram analysis use of image quality measures such as the structural similarity index manual/visual quality control	outlier detection (based on mean/median ROI values)	(see streaking and shadowing artifacts)
**Mitigation**	adjust masking, reconstruction algorithm and parameters (e.g., exclude late TEs from echo combination) use appropriate coil combination	verification of imaging parameters from DICOM header (manually or automatic) pull imaging parameters from data instead of hard coding it in the pipeline	use appropriate coil combination use of recommended acquisition protocol
**Data exclusion**	exclude if ROI affected and recalculation of the entire study cohort with the adjusted pipeline not possible (to avoid bias)		

**Table 2.** Commonly used reference regions in the literature

Reference region	advantages	disadvantages
cerebrospinal fluid^193,205,230,248–252^	automatic pipelines available^193^ no orientation dependence susceptibility of CSF unlikely to be significantly affected by disease	ventricles can be small in young subjects, resulting in segmentation inaccuracies partial volume effect because of possibly small ventricles in young subjects or compression of ventricles by pathology CSF flow artifacts choroid plexus can affect CSF susceptibility assessment in lateral ventricles
global white matter regions (not restricted to internal capsule)^28,91,253^	large region	orientation dependence might be affected by pathology, e.g. demyelination, gliosis, hemorrhage, atrophy
internal capsule^40,254–256^		orientation dependence might be affected by pathology, e.g. demyelination, gliosis, hemorrhage, atrophy, focal lesions Relatively small region
whole brain^229,236,236,257^	no extra mask required, brain mask from previous processing steps can be used intrinsic for some methods large region	might be affected by pathology and age (e.g. myelination, global demyelination, gliosis, iron accumulation, hemorrhage) due to large WM fraction similar limitations as “white matter” above.

**Table 3.** Recommendations for reporting of parameters of the acquisition hardware.

Item	Notes and examples	Recommended
Field strength	DICOM tag (0018,0087)	essential
Vendor	DICOM tag (0008, 0070)	essential
Scanner model	DICOM tag (0008, 1090)	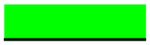
Software release	DICOM tag (0018, 1020)	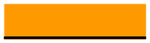
Type of coil(s) used, including information on number of channels	e.g. “… a transmitting body-coil and a 64-channel head-and-neck receiving coil”	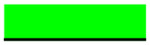
Gradient system	e.g. “… a gradient system with maximum amplitude = 50 mT/m and slew rate = 200 mT/m/ms”	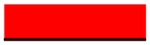

**Table 4.** Recommendations for reporting of parameters of the acquisition sequence.

Item	Notes	Recommended
Acquisition sequence type	2D *vs* 3D; GRE *vs* EPI etc.;	essential
Acquisition sequence commercial name	e.g. “SWAN”, “MERGE”, “SWlp”…	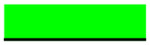
k-space sampling trajectory scheme	cartesian *vs* spiral *vs* radial etc.	essential, if not cartesian
Acquisition orientation	pure axial *vs* sagittal *vs* oblique	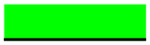
Number of echoes, TE_1_:ΔTE:TE_max_	e.g. 7 echoes, TE = 5:5:35 ms	essential
TR		essential
FA		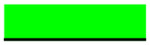
Pixel Bandwidth or Receiver Bandwidth [Hz]	DICOM tag (0018, 0095)	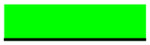
Spatial coverage (FOV) and acquisition matrix size		essential
Voxel Size	Attention: it can be different from “FOV divided by matrix size”	essential
Monopolar *vs* bipolar echoes	Indicate if the sequence produces monopolar of bipolar echoes	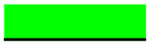
Average ± std center frequency [MHz]	In multi-scanner studies, mean ± std center frequency shall be reported for data from each scanner. For example, Siemens “3T” scanners systematically operate at <2.9T. DICOM tag (0018,0084)	essential for multi-scanner studies; unnecessary for single-scanner studies
Flow compensation	Yes / no; if yes, please indicate the compensated echo(s): all *vs* only the first one; and direction (full, phase)	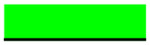
Acceleration type and factor	Yes / no. If yes: SENSE (or ASSET) vs GRAPPA (or ARC), compressed SENSE, etc.; indicate phase factor and slice factor (if 3D)	essential
Partial Fourier factor	Use should be avoided. If used, indicate partial Fourier factors in phase and slice direction	essential, if used
Partial echo (GE/Philips) *aka* Asymmetric echo (Siemens) *aka* Half echo (Hitachi)	Use should be avoided.	essential, if used
Elliptical k-space shutter	Yes / no.	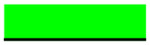
Phase stabilization	Option available only in particular implementations. If the option is available, indicate Yes / no	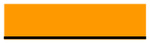 (essential if used)
Excitation pulse	Fat-sat *vs* Water-only	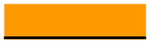
Scan duration		essential

**Table 5.** Recommendations for reporting of parameters of the reconstruction and analysis pipelines.

Item	Notes	Recommended
Toolbox used	Specify toolbox name and version (or download date), e.g. FANSI, STISuite, MEDI, etc.	mandatory
Algorithms used	For each step of the recon pipeline (phase reconstruction, echo combination, masking, phase unwrapping, background field removal, dipole inversion), please specify the algorithm used. Indicate the numerical values of relevant parameters (even if default values were used), e.g. regularization parameters.	mandatory, at least for non-default algorithms and parameters
Further processing	If further processing was necessary to make images compatible with image review environments (such as PACS) used in the study, any data manipulation (including geometrical transformations, interpolation, header data changes, etc.) should be reported	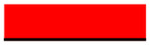
Referencing	Magnetic susceptibility values should always be reported in either ppm or ppb (parts-per-billion) and the reference region (see the Section 8) should be explicitly stated, even in the case the adopted method did implicit whole brain referencing. When the reference region used in the study is not the whole-brain mask, its [mean ± std] susceptibility value when referenced to the whole-brain mask should be reported, to enable post-hoc re-referencing for meta-analyses. Generally, it should be discussed in the Discussion section how potential pathological changes within the reference region may have biased the study outcome.	mandatory
Data inclusion/exclusion criteria	Details on data inclusion/exclusion criteria should be reported. For example: which artifacts were taken into consideration, and which level of artifact severity was considered as a threshold for inclusion/exclusion. The description of this aspect, which is study-specific, can be supported by images with representative cases in the Supplementary Materials.	Mandatory, in studies where datasets were excluded based on image quality, or when datasets with visible artifacts were deemed acceptable for inclusion
