# Supplementary material for: Recommended Implementation of Quantitative Susceptibility Mapping for Clinical Research in The Brain: A Consensus of the ISMRM Electro-Magnetic Tissue Properties Study Group
Source: ArXiv. 2023 Jul 5:arXiv:2307.02306v1. Preprint. [Version 1] (PMC10350101)
Supplement: 1 [file NIHPP2307.02306V1-supplement-1.pdf]

Supplementary Materials I

S1.1 Detailed Consensus Committee Author Contributions

This section lists the core authors of each section along with the section's lead author(s). Core authors are the members of the QSM Consensus Organization Committee who developed the initial draft of the consensus recommendations and incorporated the feedback from the QSM community (see Acknowledgement section). Lead authors orchestrated the creation of the section and are responsible for the final version. Authors are listed in alphabetical order.

Overall facilitation, organization, and coordination: Ferdinand Schweser, Yi Wang

Section "Pulse Sequences and Protocol Recommendation":
Lead authors: Jongho Lee, Jose Marques.
Core authors: Berkin Bilgic, Mauro Costagli, Christian Langkammer, Chunlei Liu, Simon Robinson, Ferdinand Schweser, Karin Shmueli, Pascal Spincemaille, Sina Straub

Section "Coil Combination, Saving and Exporting":
Lead author: Simon Robinson
Core authors: Mauro Costagli, Ferdinand Schweser, Karin Shmueli, Pascal Spincemaille

Section "Phase Unwrapping and Echo Combination":
Lead author: Xu Li
Core authors: Christian Langkammer, Chunlei Liu, Carlos Milovic, Simon Robinson, Ferdinand Schweser, Karin Shmueli

Section "Creation of Masks":
Lead author: Carlos Milovic
Core authors: Christian Langkammer, Simon Robinson, Karin Shmueli

Section "Background field removal":
Lead author: Pascal Spincemaille

Core authors: Carlos Milovic, Ferdinand Schweser

Section "Dipole Inversion":
Lead author: Yi Wang
Core authors: Xu Li, Carlos Milovic, Pascal Spincemaille

Section "Analysis of susceptibility maps":
Lead author: Sina Straub
Core authors: Mauro Costagli, Christian Langkammer, Xu Li, Ferdinand Schweser

Section "Presentation and Publication":
Lead author: Mauro Costagli
Core authors: Jeff Duyn, Christian Langkammer, Xu Li, Chunlei Liu, Ferdinand Schweser, Sina Straub

Code implementation (Supplementary Materials II):
Lead author: José Marques
Core authors: Kwok-Shing Chan

S1.2 Author Conflict of Interest Statements

In the interest of transparency, this section provides disclosures of perceived or actual risks of a conflict of interest of the authors.

Berkin Bilgic is the first author of the Wave-CAIPI publication (Bilgic et al., 2017) mentioned in Section 2.3 among the methods that provide drastic decreases in acquisition time.

Mauro Costagli has no conflicts of interest to declare.

Christian Langkammer has no conflicts of interest to declare.

Jongho Lee is the corresponding author of the papers for QSMnet, which is a deep learning-powered QSM reconstruction method (Yoon et al., 2018), and **χ**-separation, which is an advanced susceptibility mapping method for susceptibility source separation (Shin et al., 2021).

Xu Li is the first author of the paper on using both T1-weighted and susceptibility contrast multi-atlas in image analysis (Li et al., 2019), recommended in Section 8.

Chunlei Liu co-authored papers on VSHARP, Laplacian-based phase unwrapping and multi-echo weighted phase combination methods. He is a co-author of the STI Suite software and co-inventor of QSM-related patents.

José Marques is the senior author of the publication associated with the software (SEPIA) used in the Code Implementation section (Chan and Marques, 2021), and first author of the in-silico frame work to evaluate QSM reconstruction pipelines used in the QSM Challenge 2.0 (Marques et al., 2021).

Carlos Milovic is the author and manager of the FANSI toolbox, which is recommended in several sections. This includes the FANSI (Milovic et al., 2018) and Weak Harmonics (Milovic et al., 2019) algorithms, for which he is the first author.

Simon Robinson is the senior author of the coil combination methods MCPC-3D-S and ASPIRE (Eckstein et al., 2018) (Eckstein et al., 2019) which are mentioned in Section 2 as alternatives to the recommended "prescan normalize and adaptive combined". He is also the senior author of the phase unwrapping method ROMEO (Dymerska et al., 2021), which is described in Section 4 and which incorporates the steps in the "weighted echo averaging with template unwrapping" approach; one of the two recommended approaches to echo combination. He is also a co-author on two papers proposing the use of phase-based quality metrics in masking (Hagberg et al., 2022; Stewart et al., 2022) in Section 5.

Ferdinand Schweser is the first author of the original publication introducing the SHARP technique (Schweser et al., 2011). The SHARP technique is the basis of the VSHARP technique recommended in Section 6. He is the first author of a paper that introduces an L1-norm type regularization dipole inversion algorithm (Schweser et al., 2012) recommended in Section 7. He is the last author of papers on using both T1-weighted and susceptibility contrast in image analysis

(Feng et al., 2017; Hanspach et al., 2017), recommended in Section 8. He is the last author of the in-silico frame work to evaluate QSM reconstruction pipelines used in the QSM Challenge 2.0 (Marques et al., 2021). He has research support from Philips Medical Systems Nederland B.V.

Kwok-Shing Chan is the first author of the publication associated with the software (SEPIA) used in the Code Implementation section (Chan and Marques, 2021).

Karin Shmueli is an author of the phase unwrapping method ROMEO (Dymerska et al., 2021), which is described in Section 4 and which incorporates the steps in the “weighted echo averaging with template unwrapping” approach; one of the two recommended approaches to echo combination.

Pascal Spincemaille is co-author on the publications describing the PDF, LBV and MEDI methods. He is co-inventor on QSM-related patents owned by Cornell University. He is consultant for and has ownership share in MedImageMetric LLC.

Sina Straub has no conflicts of interest to declare.

Peter van Zijl has research support from Philips and technology licensed to Philips.

Yi Wang is co-author on the publications describing the PDF, LBV and MEDI methods. He is co-inventor on QSM-related patents owned by Cornell University. He is consultant for and has ownership share in MedImageMetric LLC.

S1.3 Approach and History of the Consensus Paper

The idea to create a community-driven recommendations paper for the implementation of QSM gained traction through email conversations on the email distribution list of the program committee for the 2022 Joint Workshop on MR phase, magnetic susceptibility and electrical properties mapping. One of the members of the later established QSM Consensus Organization Committee (YW) revived the idea to create a consensus or white paper for QSM, which had previously been proposed during the standardization session at the 2019 International Workshop on MRI Phase Contrast and Quantitative Susceptibility Mapping held in Seoul, Korea. The discussion quickly separated from the program committee, with the group involved growing to include 13 interested

volunteers. The first meeting for the project was organized by two members of the QSM Consensus Organization Committee (FS and YW) and occurred virtually on March 24, 2022. At this first meeting, the group agreed on the scope, title, the main paper sections, an action plan for achieving consensus and writing the paper, and a timeline. After the meeting, all participants specified which of nine paper sections they would be interested in developing for the QSM community, and whether they would consider a leading role in this process. Each section team was tasked with developing a first set of recommendation statements to be disseminated to the ISMRM Electro-Magnetic Tissue Properties Study Group as a starting point for achieving consensus. Eight sections for the paper were identified that corresponded to the segments of the QSM acquisition and processing pipeline: 1) Pulse Sequences and Protocol Recommendation, 2) Coil Combination, Saving + Exporting, 3) Phase Unwrapping and Echo Combination, 4) Masking, 5) Background field removal, 6) Inversion, 7) Analysis of susceptibility map, and 8) Presentation and Publication. Lead authors and contributing core authors for each section are listed in the Supplementary Materials Section S1.1 above.

The QSM Consensus Organization Committee reconvened on April 20, 2022 and agreed to disseminate the consensus recommendations to the QSM community for feedback at the business meeting of the ISMRM Electromagnetic Tissue Properties Study Group on June 3, 2022. The group agreed to revise the consensus recommendations and wrote, for each section, a brief overview section of the subject matter, providing a first full draft of the consensus recommendations. At the ISMRM 2022 annual meeting, a subset of the committee met in person to discuss the consensus paper project in person (May 8, 2022), and various members attended the session on white papers at the conference to better understand the requirements of such a format both regarding its preparation and the final product.

At the study group meeting on June 3, 2022, the consensus initiative was presented (FS) and a draft of the consensus recommendations was made available to the study group members as an editable online document. Study group members and non-members of all career levels with expertise in QSM were invited to contribute to the manuscript by reviewing it and suggesting modifications or extensions for the QSM Consensus Organization Committee to incorporate. The link to the online document was provided at the study group meeting and distributed after the study group meeting through the study group mailing list. After the open feedback period, the QSM Consensus Organization Committee incorporated the feedback received, discussed open questions, and, on 7/14/2022, defined the final timeline aiming for submission of the manuscript

shortly after the 2022 Joint Workshop on MR Phase, Magnetic Susceptibility and Electrical Properties Mapping in Lucca, Italy (10/16-10/19/2022). The committee revised the manuscript through several review iterations in which either only section leaders or all core-authors were involved. Some section leaders chose to perform surveys among the committee members to quantify and resolve disagreement on controversial statements or requests from the community for which the section sub-groups could not arrive at a unanimous recommendation. All surveys and their outcomes were reported in the manuscript.

The first complete version of the manuscript was provided to all participants of the 2022 Joint Workshop on MR Phase, Magnetic Susceptibility and Electrical Properties Mapping via email on 10/10/2022. The manuscript was discussed in personal communications at the workshop and a summary of the recommendations was presented in Session 9 of the workshop (10/19) followed by an open discussion with the workshop attendees. The committee incorporated the feedback collected from workshop attendees and distributed the resulting manuscript to all members of the EMTP SG via the study group mailing list on 11/23/2022 with the request to disseminate the manuscript further to interested parties and to provide feedback via email by 12/15/2022. During this period, the committee actively reached out to the industry to confirm vendor-specific statements in the manuscript and seek additional input from industry representatives on the manuscript. On 12/16, the committee held an EMTP SG Virtual Meeting which presented the final manuscript, including background information on the process employed by the committee, as well as a summary of all consensus recommendations. Between 12/16/2023 and 3/30/2023, the committee incorporated all suggestions from the October/November feedback period and finalized the manuscript for submission and official endorsement by the ISMRM EMTP study group. In addition to the study group endorsement, the committee distributed an online form to provide the opportunity of endorsement to EMTP SG non-members, listed in Section S1.4 below.

S1.4 Individuals Endorsing the Consensus Who Are Not Members of the ISMRM EMTP Study Group

In the published paper, this section will include a list of all non-members who endorsed the manuscript after the study group endorsement.

S1.5 Suggested Protocols

Please note that the protocols below are simply suggestions (with timings rounded to the nearest millisecond) and that the parameters can, of course, be adjusted where needed, according to the principles in Section 2, to accommodate differences in scanner hardware and software.

1.5T Protocol

TR = 50 ms; TE_1 = 5 ms; Echo spacing = 10 ms; Number of echoes = 5; flip angle = 23°

Matrix size (AP LR HF) = 176 x 140 x 114

Resolution = isotropic 1.2 mm

Parallel imaging factor = 2; elliptical k-space shutter

Acquisition time = 6 mins

3T Protocol

TR = 33 ms; TE_1 = 5 ms; Echo spacing = 6 ms; Number of echoes = 5; flip angle = 15°

Matrix size (AP LR HF) = 256 x 176 x 144

Resolution = isotropic 1 mm

Parallel imaging factor = 2; elliptical k-space shutter

Acquisition time = 6 mins

7T Protocol

TR = 25 ms; TE_1 = 4 ms; Echo spacing = 4 ms; Number of echoes = 5; flip angle = 10°

Matrix size (AP LR HF) = 296 x 234 x 190

Resolution = isotropic 0.75 mm

Parallel imaging factor = 3; elliptical k-space shutter

Acquisition time = 5 mins

S1.6 Resources for Susceptibility Map Analysis

Structural/ROI segmentation

- Uses QSM contrast:

- Advanced Normalization Tools - ANTs (https://github.com/ANTsX/ANTs)
- SuscEptibility mapping PIpeline tool for phAse images – SEPIA (https://github.com/kschan0214/sepia) (uses ANTs for atlas-based segmentation)
- https://mricloud.org/
- STI Suite (https://people.eecs.berkeley.edu/~chunlei.liu/software.html)

- Not QSM-specific/-based, uses T1-weighted images:

- Fastsurfer (https://github.com/Deep-MI/FastSurfer)
- Neurodesk (https://github.com/NeuroDesk) (ROI segmentation is based on T1-weighted images, although it includes ANTs)
- QSMxT (https://github.com/QSMxT/QSMxT) (ROI segmentation is based on T1-weighted images, although it includes ANTs)
- Freesurfer (https://surfer.nmr.mgh.harvard.edu/)
- FSL (https://fsl.fmrib.ox.ac.uk/fsl/fslwiki)
- SPM12 (http://www.fil.ion.ucl.ac.uk/spm/software/spm12/)
- A Computational Anatomy Toolbox for SPM – CAT (https://neuro-jena.github.io/cat/)

Lesion segmentation

- Uses QSM contrast:

- QSMRim-Net (https://github.com/tinymilky/QSMRim-Net)

- Not QSM-specific/-based, uses T1-weighted images:

- LST - A lesion segmentation tool for SPM (https://www.applied-statistics.de/lst.html)
- NicMSlesions (https://github.com/sergivalverde/nicMSlesions/)
- Freesurfer (https://surfer.nmr.mgh.harvard.edu/fswiki/Samseg)

QSM-based vessel segmentation

- Uses QSM contrast:

- CVI-MRI (https://github.com/philgd/CVI-MRI)

- Nighres (https://github.com/nighres/nighres)
- GRE_vessel_seg (https://github.com/SinaStraub/GRE_vessel_seg)

Voxel based analysis

- Uses QSM contrast:

- Advanced Normalization Tools - ANTs (https://github.com/ANTsX/ANTs)

- Not QSM-specific/-based, uses T1-weighted images:

- Freesurfer (https://surfer.nmr.mgh.harvard.edu/)
- FSL (https://fsl.fmrib.ox.ac.uk/fsl/fslwiki)
- SPM12 (http://www.fil.ion.ucl.ac.uk/spm/software/spm12/)
- A Computational Anatomy Toolbox for SPM – CAT (https://neuro-jena.github.io/cat/)

References

Bilgic, B., Ye, H., Wald, L.L., Setsompop, K., 2017. Simultaneous Time Interleaved MultiSlice (STIMS) for Rapid Susceptibility Weighted acquisition. NeuroImage 1–10. https://doi.org/10.1016/j.neuroimage.2017.04.036

Chan, K.-S., Marques, J.P., 2021. SEPIA—Susceptibility mapping pipeline tool for phase images. NeuroImage 227, 117611. https://doi.org/10.1016/j.neuroimage.2020.117611

Dymerska, B., Eckstein, K., Bachrata, B., Siow, B., Trattnig, S., Shmueli, K., Robinson, S.D., 2021. Phase unwrapping with a rapid opensource minimum spanning tree algorithm (ROMEO). Magn. Reson. Med. 85, 2294–2308. https://doi.org/10.1002/mrm.28563

Eckstein, K., Dymerska, B., Bachrata, B., Bogner, W., Poljanc, K., Trattnig, S., Robinson, S.D., 2018. Computationally Efficient Combination of Multi-channel Phase Data From Multi-echo Acquisitions (ASPIRE): Combination of Multi-Channel Phase Data from Multi-Echo Acquisitions (ASPIRE). Magn. Reson. Med. 79, 2996–3006. https://doi.org/10.1002/mrm.26963

Feng, X., Deistung, A., Dwyer, M.G., Hagemeier, J., Polak, P., Lebenberg, J., Frouin, F., Zivadinov, R., Reichenbach, J.R., Schweser, F., 2017. An improved FSL-FIRST pipeline for subcortical gray matter segmentation to study abnormal brain anatomy using

quantitative susceptibility mapping (QSM). Magn Reson Imaging 39, 110–122. https://doi.org/10.1016/j.mri.2017.02.002

Hagberg, G.E., Eckstein, K., Tuzzi, E., Zhou, J., Robinson, S., Scheffler, K., 2022. Phase-based masking for quantitative susceptibility mapping of the human brain at 9.4T. Magn. Reson. Med. 88, 2267–2276. https://doi.org/10.1002/mrm.29368

Hanspach, J., Dwyer, M.G., Bergsland, N.P., Feng, X., Hagemeier, J., Bertolino, N., Polak, P., Reichenbach, J.R., Zivadinov, R., Schweser, F., 2017. Methods for the computation of templates from quantitative magnetic susceptibility maps (QSM): Toward improved atlas- and voxel-based analyses (VBA). J Magn Reson Imaging 46, 1474–1484. https://doi.org/10.1002/jmri.25671

Li, X., Chen, L., Kutten, K., Ceritoglu, C., Li, Y., Kang, N., Hsu, J.T., Qiao, Y., Wei, H., Liu, C., Miller, M.I., Mori, S., Yousem, D.M., van Zijl, P.C.M., Faria, A.V., 2019. Multi-atlas tool for automated segmentation of brain gray matter nuclei and quantification of their magnetic susceptibility. NeuroImage 191, 337–349. https://doi.org/10.1016/j.neuroimage.2019.02.016

Marques, J.P., Meineke, J., Milovic, C., Bilgic, B., Chan, K., Hedouin, R., Zwaag, W., Langkammer, C., Schweser, F., 2021. QSM reconstruction challenge 2.0: A realistic in silico head phantom for MRI data simulation and evaluation of susceptibility mapping procedures. Magn. Reson. Med. 86, 526–542. https://doi.org/10.1002/mrm.28716

Milovic, C., Bilgic, B., Zhao, B., Acosta-Cabronero, J., Tejos, C., 2018. Fast nonlinear susceptibility inversion with variational regularization. Magn. Reson. Med. 80, 814–821. https://doi.org/10.1002/mrm.27073

Milovic, C., Bilgic, B., Zhao, B., Langkammer, C., Tejos, C., Acosta-Cabronero, J., 2019. Weak-harmonic regularization for quantitative susceptibility mapping. Magn. Reson. Med. 81, 1399–1411. https://doi.org/10.1002/mrm.27483

Schweser, F., Deistung, A., Lehr, B.W., Reichenbach, J.R., 2011. Quantitative imaging of intrinsic magnetic tissue properties using MRI signal phase: An approach to in vivo brain iron metabolism? NeuroImage 54, 2789–2807. https://doi.org/10.1016/j.neuroimage.2010.10.070

Schweser, F., Sommer, K., Deistung, A., Reichenbach, J.R., 2012. Quantitative susceptibility mapping for investigating subtle susceptibility variations in the human brain. NeuroImage 62, 2083–2100. https://doi.org/10.1016/j.neuroimage.2012.05.067

Shin, H.-G., Lee, Jingu, Yun, Y.H., Yoo, S.H., Jang, J., Oh, S.-H., Nam, Y., Jung, S., Kim, S., Fukunaga, M., Kim, W., Choi, H.J., Lee, Jongho, 2021. χ-separation: Magnetic

susceptibility source separation toward iron and myelin mapping in the brain. NeuroImage 240, 118371. https://doi.org/10.1016/j.neuroimage.2021.118371

Stewart, A.W., Robinson, S.D., O'Brien, K., Jin, J., Widhalm, G., Hangel, G., Walls, A., Goodwin, J., Eckstein, K., Tourell, M., Morgan, C., Narayanan, A., Barth, M., Bollmann, S., 2022. QSMxT: Robust masking and artifact reduction for quantitative susceptibility mapping. Magn. Reson. Med. 87, 1289–1300. https://doi.org/10.1002/mrm.29048

Yoon, J., Gong, E., Chatnuntawech, I., Bilgic, B., Lee, Jingu, Jung, W., Ko, J., Jung, H., Setsompop, K., Zaharchuk, G., Kim, E.Y., Pauly, J., Lee, Jongho, 2018. Quantitative susceptibility mapping using deep neural network: QSMnet. NeuroImage 179, 199–206. https://doi.org/10.1016/j.neuroimage.2018.06.030

Supplementary Materials II – Example Data and Code for QSM Reconstruction

Introduction

These supplementary materials provide an overview of the QSM reconstruction processing, from scanner-provided data to QSM maps, based on the recommendations present in the main text. This document has two main purposes:

(1) Allowing readers to reproduce the results shown throughout the paper, and

(2) Providing readers with the means to reconstruct their own data using the recommended processing with data acquired on any of the 3 major MR providers broadly following the recommendations.

Full datasets, results and processing scripts are available on Zenodo:
https://doi.org/10.5281/zenodo.7410455
Example data of version v0.2.1 were used in this paper.

S2.1 Data availability

Data are available from scanners of three vendors: GE, SIEMENS, and PHILIPS, acquired with the recommended protocol described in Section 2 and Supplementary Materials I, Section S1.5. For each vendor, both monopolar and bipolar readout strategies were used to acquire the data for demonstration purposes. The data from GE and SIEMENS scanners were not pre-scan normalized (which does not follow the recommendations), while the PHILIPS data have two normalization methods applied. In

this way, we demonstrate the robustness of the proposed pipeline to a variety of implementations of the recommended protocol.

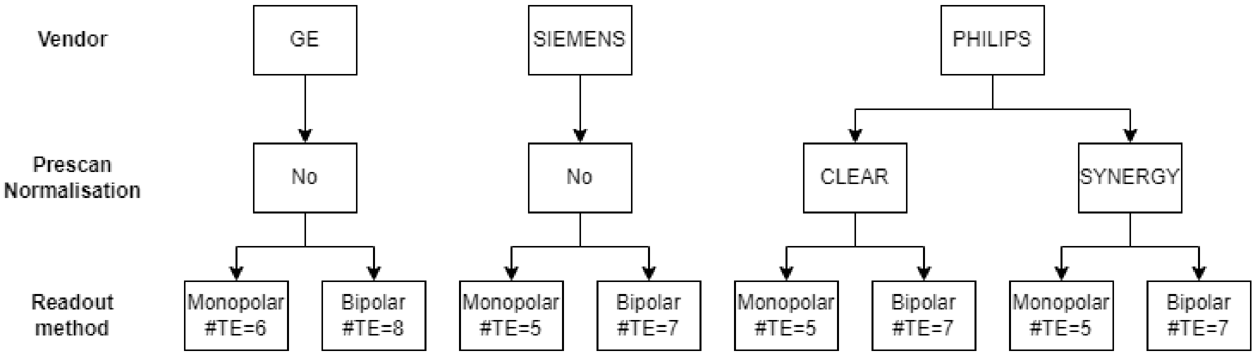

Figure S2.1: An illustration of the raw data available.

S2.2 Data preparation and organization

Background

There are two zip-files available in Zenodo containing the example data organised in two different ways:

(a) “QSM_CONSENSUS_Paper_Example_DICOM_code.zip”
This zip file contains example DICOM images exported from the scanners without any modifications. The code directory accompanied with this file contains the scripts to (1) convert the DICOM images to NIFTI format, (2) organise the NIFTI images according to BIDS v1.8.0, and (3) perform QSM reconstruction.

(b) “QSM_Consensus_Paper_example_Data_Result_Code.zip”
This zip file contains all data and results that were produced by running all the scripts provided in the code directory.

The following Data Preparation section provides information on all the pre-processing steps to prepare the unmodified DICOM images to the BIDS format data that is ready for

QSM reconstruction in SEPIA. If the readers are interested in the QSM reconstruction and work on the “QSM_Consensus_Paper_example_Data_Result_Code.zip” file only, they may skip Section “Data Preparation”.

Data Preparation

The scripts created for this section were tested on a Mac system (macOS 13.2) and a Linux system (CentOS 7). They were not tested on Windows systems and would require adaptations to work there.

Most imaging software in the field typically deals with images in Analyze or NIfTI format. As such the raw data (imaging data after coil combination) provided has to be converted to this format in 4 steps:

Step 1: Unzip the received data and reformat the directory structure
Script: Preparation_01_rename_received_data.sh

Step 2: Convert DICOM images into NIfTI format
Dependency: dcm2niix (version 1.0.20220720)
Script: Preparation_02_convert_dicom2nii.sh

Step 3: Rename the files according to the BIDS format (Brain Imaging Data structure)
Dependency: Matlab R2016b onwards
The naming strategy is as follows:

- Vendors are identified using the session tag: **ses-<GE|PHILIPS|SIEMENS>**
- For **GE** and **SIEMENS**, different readout methods are identified using the acquisition tag: **acq-<Bipolar|Monopolar>**;
- For **PHILIPS**, the normalisation method is also printed on the acquisition tag, i.e., **acq-<BipolarCLEAR|BipolarSYNERGY|MonopolarCLEAR|MonopolarSYNERGY>**

Script: Preparation_03_rename_to_bids_format.m

Step 4: Prepare NIFTI data for SEPIA

Dependency: (1) Matlab R2016b onwards, (2) SEPIA v1.2.2.4

Involves the following operation:

- Combining individual multi-echo 3D volumes into a single 4D volume with TE in the 4th dimension;
- Obtaining header info (e.g., B_0 direction and TE) from NIfTI header and JSON sidecar files and saving as SEPIA's header format;
- (GE only) Correcting inter-slice opposite polarity on real and imaginary images and exporting phase images from the corrected real/imaginary data

Script: Preparation_04_prepare_for_sepia.m

Data organization

The following tree diagram illustrates the directory structure of how the data are organised after running all the scripts provided in the code directory "QSM_Consensus_Paper_Example_Code/". The content of the different directories is mentioned after the comment "%" symbol. Note that similar directories exist under the "/derivatives/SEPIA/SIEMENS/" and "/derivatives/SEPIA/PHILIPS/" as under "/derivatives/SEPIA/GE/".

```
QSM_Consensus_Paper_Example_DICOM_Code/
|-- QSM_CONSENSUS_DATA.zip                % Zip file containing all unmodified DICOM images
|-- protocols                             % Protocol text/HTML files
|-- QSM_Consensus_Paper_Example_Code      % Containing all the scripts
|   |-- doc                               % Containing manual to use the Example data
|   |-- From_DICOM_zip_file_to_SEPIA_ready  % Scripts for preparing QSM_CONSENSUS_DATA.zip
|   |-- SEPIA_Pipeline_FANSI              % SEPIA pipeline config files with FANSI recon
|   `-- SEPIA_Pipeline_MEDI               % SEPIA pipeline config files with MEDI recon
|-- raw                                   % DICOM images
|-- converted                             % dcm2niix output
|   |-- GE
|   |   |-- Bipolar                       % Bipolar readout acquisition
|   |   `-- Monopolar                     % Monopolar readout acquisition
|   |-- PHILIPS
|   |   |-- Bipolar_CLEAR                 % with CLEAR normalisation
|   |   |-- Bipolar_SYNERGY               % with SYNERGY normalisation
|   |   |-- Monopolar_CLEAR
```

```
|   |   `-- Monopolar_SYNERGY
|   `-- SIEMENS
|       |-- Bipolar
|       `-- Monopolar
`-- derivatives                             % directory contains all derived output
    `-- SEPIA                               % SEPIA output
        |-- GE
        |   |-- Bipolar
        |   |   `-- GRE
        |   |       |-- Pipeline_FANSI      % Full QSM recon using FANSI for dipole inversion
        |   |       `-- Pipeline_MEDI       % Full QSM recon using MEDI for dipole inversion

        |   `-- Monopolar
        |       `-- GRE
        |           |-- Pipeline_FANSI      % Full QSM recon using FANSI for dipole inversion
        |           `-- Pipeline_MEDI       % Full QSM recon using MEDI for dipole inversion
        |-- PHILIPS
        `-- SIEMENS
```

2.3 QSM reconstruction pipeline

This section describes all the QSM reconstruction processing steps performed in SEPIA. All the processing steps are specified in the SEPIA pipeline configuration files, which are in the sub-directories of the script directory: *'QSM_Consensus_Paper_Example_Code/SEPIA_Pipeline_FANSI/'* and *'QSM_Consensus_Paper_Example_Code/SEPIA_Pipeline_MEDI/'*, corresponding to the two processing pipelines demonstrated as follows.

Environment and dependencies

The data were processed using the following set-up:

Operating system:

- Linux CentOS 7

Environment:

- Matlab R2021a (but the scripts are backwards compatible with earlier Matlab versions from R2016b to R2022a)

Dependencies:

The following QSM toolboxes have to be downloaded and integrated into SEPIA following the instruction provided on the SEPIA documentation website (https://sepia-documentation.readthedocs.io/en/latest/getting_started/Installation.html):

- SEPIA v1.2.2.4 (https://github.com/kschan0214/sepia/releases/tag/v1.2.2.4)
- MRITOOLS v3.5.6 (https://github.com/korbinian90/CompileMRI.jl/releases/tag/v3.5.6)
- MEDI toolbox (release: 15th January 2020) (http://pre.weill.cornell.edu/mri/pages/qsm.html)
- FANSI toolbox [v3] (https://gitlab.com/cmilovic/FANSI-toolbox)
- STI Suite v3.0 (https://people.eecs.berkeley.edu/~chunlei.liu/software.html)

QSM reconstruction using Example data

This section describes all the QSM reconstruction settings that were used on the example data. All the methods and algorithm parameters mentioned were already specified in the SEPIA pipeline configuration files (sepia_<GE|PHILIPS|SIEMENS>_<Monopolar|Bipolar>_config.m), which can be found in the sub-directories of the code folder "QSM_Consensus_Paper_Example_Code/": *"SEPIA_Pipeline_FANSI/"* and *"SEPIA_Pipeline_MEDI/"*. Here, we provide an overview of the main parameters of each of these pipelines (Tables S2.1-S2.4) for the readers' convenience.

Processing steps

Step 1: Preparation

- (GE only) Phase data must be inverted before QSM reconstruction processing (i.e., phase = -phase), so that paramagnetic susceptibility gives a positive value while diamagnetic susceptibility gives a negative value, same as the data from other vendors. This step was performed with the option provided by SEPIA.
- Brain mask is obtained by using MEDI toolbox implementation of FSL's BET on the 1st echo magnitude image, using default setting -f 0.5 -g 0

- (Bipolar readout data only) Bipolar readout correction based on (Li et al., 2015) using the implementation provided with SEPIA.
- Note that the relevant sequence parameters such as echo time and slice orientation are automatically derived from the data.

Step 2: Total field estimation and echo combination

Table S2.1: Algorithm parameters for total field estimation and echo combination.

Parameters	Values	Remark
**Echo phase combination**	ROMEO total field calculation	(Dymerska et al., 2020)
**MCPC-3D-S phase offset correction**	On	
**Mask for unwrapping**	SEPIA mask	FSL's BET mask
**Using ROMEO Mask in SEPIA**	Off	
**Exclude voxel using relative residual with threshold**	0.3 (applied on weighting map)	See https://sepia-documentation.readthedocs.io/en/latest/method/weightings.html

Step 3: Background field removal

Table S2.2: Algorithm parameters for background field removal.

Parameters	Values	Remark
**Method**	VSHARP	(Li et al., 2011); SEPIA's implementation
**Maximum spherical mean value filtering size**	12	Unit: voxels
**Minimum spherical mean value filtering size**	1	Unit: voxels
**Remove residual B1 field**	No	
**Erode brain mask before BFR**	1	Unit: voxel

**Erode brain mask after BFR**	0	

<u>**Step 4: Dipole inversion**</u>

We demonstrate the dipole inversion steps with two recommended methods (FANSI and MEDI).

Step 4.1: FANSI dipole inversion

Table S2.3: Algorithm parameters for dipole field inversion using 'SEPIA_PIPELINE_FANSI' pipeline.

**Parameters**	**Values**	**Remark**
**Method**	FANSI	(Milovic et al., 2019, 2018)
**Iteration tolerance**	0.1	
**Maximum number of iterations**	400	
**Gradient L1 penalty, regularisation weight**	0.0005	
**Gradient consistency weight**	0.05	
**Fidelity consistency weight**	1	
**Solver**	Non-linear	
**Constraint**	TV	
**Method for regularisation spatially variable weight**	Vector field	
**Using weak harmonic regularisation**	On	
**Harmonic constraint weight**	150	

**Harmonic consistency weight**	3	
**Reference tissue**	Brain mask	

Step 4.2: MEDI dipole inversion

Table S2.4: Algorithm parameters for dipole field inversion using 'SEPIA_PIPELINE_MEDI' pipeline.

**Parameters**	**Values**	**Remark**
**Method**	MEDI	(Liu et al., 2011)
**Regularisation parameter (lambda)**	2000	
**Method of data weighting**	1	SNR weighting
**Percentage of voxels considered to be edges**	90	
**Array size for zero padding**	[0 0 0]	
**Performing spherical mean value operator**	On	
**Radius of the spherical mean value operation**	5	Unit: voxel
**Performing modal error reduction through iterative tuning (MERIT)**	On	
**Performing automatic zero reference (MEDI+0)**	Off	
**Reference tissue**	Brain mask	

Adaptation of the example pipeline to other studies

The provided SEPIA pipeline configuration file (sepia_<GE|PHILIPS|SIEMENS>_<Monopolar|Bipolar>_config.m) can be reused for

other studies, assuming the data in these studies have the compatible input directory described in the SEPIA documentation website (https://sepia-documentation.readthedocs.io/en/latest/getting_started/Data-preparation.html):

This can be done by updating the “input” variable in the configuration file to the location of the input directory that contains all the essential data in your computer. Alternatively, if a graphical operation is preferred, the SEPIA pipeline configuration files can be imported to the SEPIA’s GUI by using the “Load config” button on the bottom left of the GUI display and then select the configuration .m file. The GUI will then be updated to the specified methods and algorithm parameters according to the text in the configuration file. Readers can then specify the required input and output information on the “I/O” panel on the GUI.

2.4 Example results

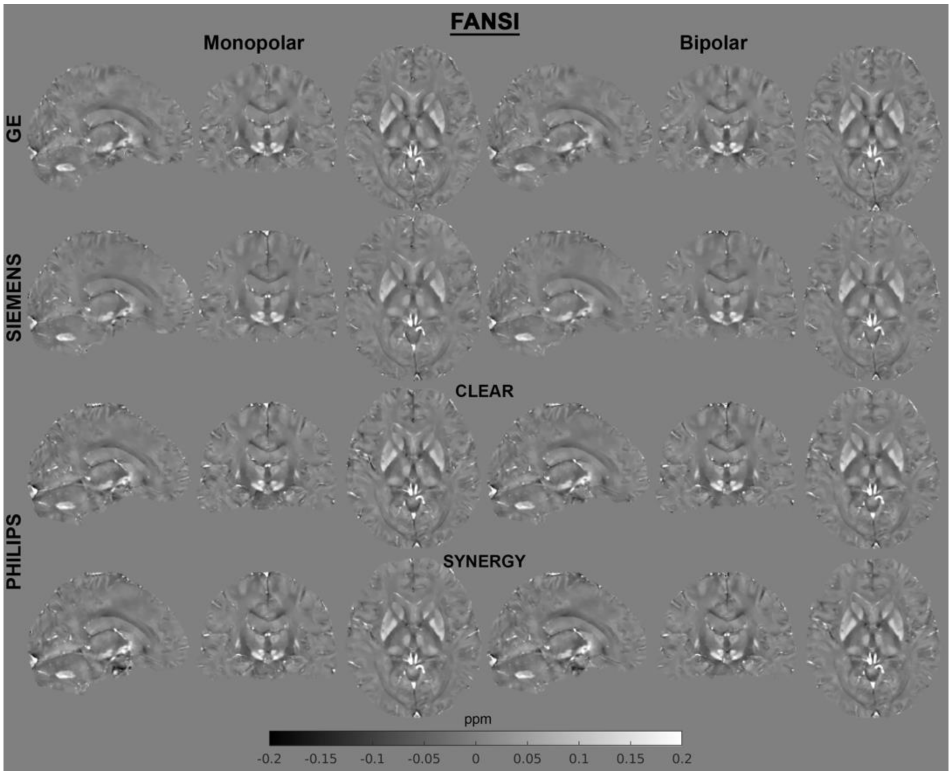

Figure S2.2: Susceptibility maps derived using the “SEPIA_Pipeline_FANSI” processing pipeline.

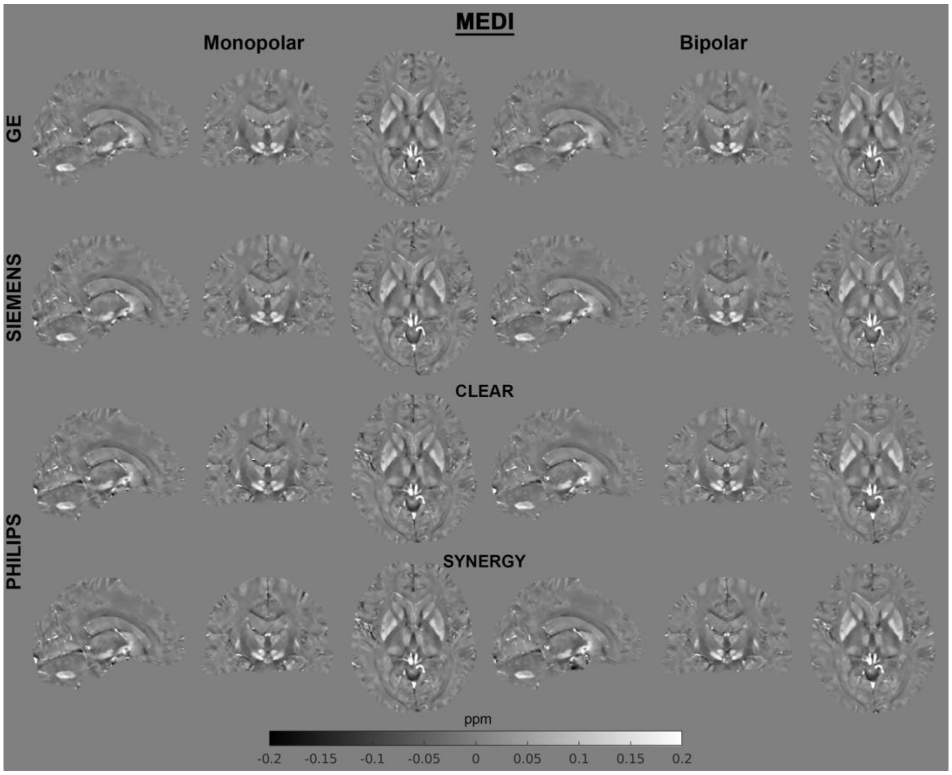

Figure S2.3: Susceptibility maps derived using the “SEPIA_Pipeline_MEDI” processing pipeline.

References

Dymerska, B., Eckstein, K., Bachrata, B., Siow, B., Trattnig, S., Shmueli, K., Robinson, S.D., 2021. Phase unwrapping with a rapid opensource minimum spanning tree algorithm (ROMEO). Magnetic resonance in medicine 85, 2294–2308. https://doi.org/10.1002/mrm.28563

Karsa, A., Punwani, S., Shmueli, K., 2020. An optimized and highly repeatable MRI acquisition and processing pipeline for quantitative susceptibility mapping in the head-and-neck region. Magnetic resonance in medicine 84, 3206–3222. https://doi.org/10.1002/mrm.28377

Li, J., Chang, S., Liu, T., Jiang, H., Dong, F., Pei, M., Wang, Q., Wang, Y., 2015. Phase-corrected bipolar gradients in multi-echo gradient-echo sequences for quantitative susceptibility mapping. Magma (New York, N.Y.) 28, 347–355. https://doi.org/10.1007/s10334-014-0470-3

Li, W., Wu, B., Liu, C., 2011. Quantitative susceptibility mapping of human brain reflects spatial variation in tissue composition. Neuroimage 55, 1645–1656. https://doi.org/10.1016/j.neuroimage.2010.11.088

Liu, T., Liu, J., Rochefort, L. de, Spincemaille, P., Khalidov, I., Ledoux, J.R., Wang, Y., 2011. Morphology enabled dipole inversion (MEDI) from a single-angle acquisition: Comparison with COSMOS in human brain imaging. Magnetic resonance in medicine 66, 777–783. https://doi.org/10.1002/mrm.22816

Milovic, C., Bilgic, B., Zhao, B., Acosta-Cabronero, J., Tejos, C., 2018. Fast nonlinear susceptibility inversion with variational regularization. Magnetic resonance in medicine 80, 814–821. https://doi.org/10.1002/mrm.27073

Milovic, C., Bilgic, B., Zhao, B., Langkammer, C., Tejos, C., Cabronero, J.A., 2019. Weak-harmonic regularization for quantitative susceptibility mapping. Magnetic resonance in medicine 81, 1399–1411. https://doi.org/10.1002/mrm.27483

Schweser, F., Deistung, A., Sommer, K., Reichenbach, J.R., 2013. Toward online reconstruction of quantitative susceptibility maps: superfast dipole inversion. Magnetic resonance in medicine 69, 1582–1594. https://doi.org/10.1002/mrm.24405

Supplementary Materials III - Saving Phase Data and Generating Combined Phase Images: Detailed Information for MR Manufacturers and Systems

Siemens

“Adaptive-combined with prescan normalize” (AC-PN): This is the recommended approach for systems up to 3T with modern software versions - VE onwards (Jellus V and Kannengiesser S, 2014).
Availability: software version VE11 and later, systems up to 3T.
How to: In the product GRE sequence, i) in the System>Miscellaneous tab, set Coil Combine Mode to Adaptive Combined, ii) in the Resolution>Filter Image tab, check the Prescan Normalize box and iii) in the Contrast>Dynamic tab, set Reconstruction to Magn./Phase.
Limitations: No acceleration is possible in the second phase-encode direction. Not compatible with the SWI option (checkbox on the overview tab).

“Singular Valued Decomposition Phase Combination”: An alternative phase combination provided by Siemens for UHF systems, also applicable to single-echo acquisitions. (Inati et al., 2014)
Availability: software version VE11 and later (for research purposes only).
How to: acquire GRE data and retro-reconstruct using the tool TWIX (as advanced user, Windows>Run>twix), changing the ICE program (tICEProgramName) from %SiemensIceProgs%\IceProgram3D to %SiemensIceProgs%\IcePat and set YAPS.AdaptiveCoilCombineAlgo (default -1 -> ACC_ALGO_EVD_PSNPC = 9) to ACC_ALGO_EVD_SVDPC = 5. Does not to work with "meas dependencies" on unless using the "retro recon UI tool" at VE (echo symbol) / NX.

Limitations: None known. For research purposes only. Needs Advanced User privileges.

“ASPIRE Online”: A coil combination method for multi-echo data, suitable for systems without a body coil, e.g. UHF (Eckstein et al. 2018).
Availability: VB17, VE11C, VE12U, all field strengths, via C2P (simon.robinson@meduniwien.ac.at).
How to: In the C2P GRE sequence (ke_gre_aspire_*), in the Sequence>Special tab, set Phase Combination to ASPIRE.
Features: Allows acceleration in the second phase-encode direction and provides T2*/R2* mapping.
Limitations: Requires at least two echoes and TE2=2*TE1. For bipolar, requires at least three echoes and TE3=3*TE1, TE2=2*TE1. Needs Advanced User privileges for installation.

“MCPC-3D-S”/“ASPIRE Offline”: A coil combination method for multi-echo data, suitable for systems without a body coil, e.g. UHF (Eckstein et al. 2018).
Availability: All systems.
How to: This method needs phase and magnitude data from all channels to be saved and exported for offline processing. In the GRE sequence, i) in the System>Miscellaneous tab, check the Save uncombined box and ii) in the Contrast>Dynamic tab, set Reconstruction to Magn./Phase. Reconstruct phase and magnitude offline using https://github.com/korbinian90/ASPIRE (MATLAB) or the compiled/Julia unwrapping program ROMEO (https://github.com/korbinian90/ROMEO), which will unwrap and also combine data over coils if there is a 5th dimension (x,y,z,echo,coil).
Features: Allows acceleration in the second phase-encode direction, not subject to the echo time constraints of ASPIRE.
Limitations: Requires export of separate channel data (a large number of files).

“Virtual Reference Coil”: A coil combination method suitable for systems without a body coil, e.g. UHF (Parker et al. 2014).
Availability: All systems.

How to: This method can be performed online, using a dedicated ICE program which is available via C2P (request to Mathieu Santin; mathieu.santin@icm-institute.org) or offline, in which case phase and magnitude data from all channels need to be saved and exported for offline processing. In the GRE sequence, i) in the System>Miscellaneous tab, check the Save uncombined box and ii) in the Contrast>Dynamic tab, set Reconstruction to Magn./Phase. Reconstruct phase and magnitude offline using https://github.com/mckib2/virtcoilphase.
Features: Allows acceleration in the second phase-encode direction, applicable to single-channel data.
Limitations: Requires export of separate channel data (a large number of files). Can fail in the cerebellum, for large objects or at field strengths above 7T. Needs Advanced User privileges for installation (online version).

"Multi-echo Coil Combination": A coil combination method for multi-echo data, suitable for 3T and 7T systems.
Availability: VB17 via C2P (pas2018@med.cornell.edu).
How to: In the C2P GRE sequence (customer/gre), set Coil Combination to Adaptive Combine.
Features: The sequence produces suitable magnitude and phase DICOM data directly on the scanner. Before channel combination, the phase of the first echo is subtracted from the phase of all echoes after which the channel phases are averaged to obtain the combined phase (Eq 13 in Bernstein et al., 1994). The magnitude is obtained using sum-of-squares.
Limitations: 1) The method only works for 2 or more echoes. 2) The image reconstruction method is memory intensive. Needs Advanced User privileges for installation.

Philips

"SENSE or CS-SENSE": The product 3D FFE sequence allows reconstruction of coil-combined magnitude/phase/real/imaginary data using SENSE or CS-SENSE (compressed-sensing)

Availability: SENSE is available in all systems with V4 and later software versions, CS-SENSE is available in some V5 systems

How to: in the "Postproc" tab -> "Images" -> Select output of "M" and "P" for magnitude and phase output. For conversion with DCM2NII and DCM2NIIX, the "Philips precise scaling" parameter should be set to ON to avoid using the other rescaling factors provided (which only adjust relative pixel intensity but do not provide quantitative rescaled values).

When using the SWIp product sequence (e.g. the clinic, to get SWI images), save the magnitude and phase data using the "Delayed reconstruction" procedure (need to turn "Postproc" tab -> "Save raw data" to "yes"). On Release 5 of the software (R5), this does not require a research key and is performed by right-clicking on the exam card and selecting the delayed recon option.

Limitations: For V5, need to set "Postproc" tab -> "Images" -> "SWIp" to "no" to allow unfiltered phase output for QSM, otherwise have to use the "Delayed reconstruction".

GE

"Research sequence": The product SPGR sequence allows the reconstruction of mag/real/imag images by enabling the right features in the source code. This requires a research key.

Availability: The Cornell group (pas2018@med.cornell.edu) can share a compiled version with groups with a valid GE research license (RCSL). Software version 14 and newer.

How to: The sequence allows approximating the recommended protocol (precise TE/TR will be scanner dependent). ASSET is required to obtain correct phase, as is the disabling of any image filter and 3D geometry correction. The default 2D gradient correction works fine. Use TE = minFull. Phase Image = OFF. Must appropriately set CVs rhfiesta, rhrcctrl and rhrcxres (to the acquired matrix size).

Limitations: precise TE/TR will be scanner dependent. Export of separate channel phase and magnitude images requires a research key.

“**Product sequence**”: The product SWAN and MERGE sequences allow reconstructing mag/real/imag by following the steps indicated below. MERGE allows shorter TEs and TRs. To modify the necessary CVs, a research key is required.
Availability: SWAN and MERGE are commercial sequences provided by the vendor.
How to: The sequence allows approximating the recommended protocol (precise TE/TR will be scanner dependent). ASSET (or ARC) is required to obtain correct phase, and TE must be set to “minFull”. It is mandatory to set Phase Image = OFF in the GUI. The following CVs must be set as follows (research key required): rhfiesta=0 to keep echoes separated; rhrcctrl=13 to produce also the real and imaginary parts. For SWAN only, on DV25 onwards, the first echo can be imposed from the “Advanced” tab in the GUI and can be set as an integer value (CV16).
Limitations: No direct control on all TEs and TR.

Gradient nonlinearities on older systems (e.g. Signa HDx) lead to distortions when images are reconstructed with FFT from k-space data. These need to be corrected with the spherical harmonics of the gradient system (which are stored on the hard drive for GE).

References

Bernstein MA, Grgic M, Brosnan TJ, Pelc NJ. Reconstructions of phase contrast, phased array multicoil data. Magn Reson Med. 1994 Sep;32(3):330-4. doi: 10.1002/mrm.1910320308. PMID: 7984065.

Eckstein, Korbinian, Barbara Dymerska, Beata Bachrata, Wolfgang Bogner, Karin Poljanc, Siegfried Trattnig, and Simon Daniel Robinson. 2018. “Computationally Efficient Combination of Multi-Channel Phase Data From Multi-Echo Acquisitions (ASPIRE).” Magnetic Resonance in Medicine 79 (6): 2996–3006.

S. J. Inati, M. S. Hansen, and P. Kellman. A Fast Optimal Method for Coil Sensitivity Estimation and Adaptive Coil Combination for Complex Images. In ProcIntl Soc Mag Reson Med, Milan, Italy, May 2014. Abstract 4407

Jellus V and Kannengiesser S. Adaptive Coil Combination Using a Body Coil Scan as Phase Reference. In Proceedings of the 23th Annual Meeting of the ISMRM, Milan, Italy, 2014. #4406.

Parker, Dennis L., Allison Payne, Nick Todd, and J. Rock Hadley. 2014. “Phase Reconstruction from Multiple Coil Data Using a Virtual Reference Coil.” Magnetic Resonance in Medicine 72 (2): 563–69.
